# Photodynamic Therapeutic Effect of Nanostructured Metal Sulfide Photosensitizers on Cancer Treatment

**DOI:** 10.1186/s11671-022-03674-8

**Published:** 2022-03-08

**Authors:** Daysi Diaz-Diestra, Hanna Madadi Gholipour, Marjan Bazian, Bibek Thapa, Juan Beltran-Huarac

**Affiliations:** 1 Department of Chemistry, University of Puerto Rico, San Juan, PR 00931 USA; 2 Present Address: NAMSA, 400 US Highway 169 S, Suite 500, Minneapolis, MN 55426 USA; 3 Department of Physics, Babol Noshirvani University of Technology, 47148 Babol, Iran; 4 Department of Physics, Alzahra University, 19938 Tehran, Iran; 5 Advanced Imaging Research Center, University of Texas Southwestern Medical Center, Dallas, TX 75390 USA; 6 Department of Physics, Howell Science Complex, East Carolina University, Greenville, NC 27858 USA

**Keywords:** Photodynamic therapy, Metal sulfides, Photosensitizers, Cancer treatment

## Abstract

Photodynamic therapy (PDT) utilizes photosensitizers (PSs) to produce reactive oxygen species (ROSs) upon irradiation, which causes the shutdown of vessels and deprives the tumor of nutrients and oxygen, and in turn induces adverse effects on the immune system. However, significant efforts are needed to increase the efficiency in PDT in terms of light delivery to specific PSs for the clinical treatment of tumors located deep under the skin. Even though PDT offers a disease site-specific treatment modality, current efforts are directed to improve the solubility (in body fluids and injectable solvents), photostability, amphiphilicity (for tissue penetration), elimination, and systemic toxicity of traditional PSs based on porphyrin derivatives. Nanostructured materials show promising features to achieve most of such combined efforts. They can be artificially engineered to carry multiple theranostic agents onto targeted tumor sites. However, recent studies on photosensitive Cd-based nanostructures, mostly used in PDT, indicate that leeching of Cd^2+^ ions is stimulated when they are exposed to harsh biological conditions for continuous periods of time, thus making them acutely toxic and hindering their applications in in vivo settings. Since nanostructured materials are not completely immune to degradation, great strides have been made to seek new alternatives. In this review, we focus on the latest advances of Cd-free nanostructured metal transition sulfides (MTSs) as alternative PSs and study their high-energy transfer efficiency, rational designs, and potential applications in cancer-targeted PDT. Nanostructured MTSs are discussed in the context of their versatility to serve as phototherapy agents and superior properties, including their strong absorption in the NIR region, excellent photothermal conversion efficiency, controlled reactive oxygen species (ROS) production, versatile surface chemistry, high fluorescence, and structural and thermal stability. We discuss the latest advancements in correlating the self-aggregation of MTSs with their passive tumor cell targeting, highlighting their ability to efficiently produce ROSs, and mitigating their dark toxicity through polymeric functionalization. Treatment of deep-seated tumors by using these PSs upon preferential uptake by tumor tissues (due to the enhanced permeability and retention effect) is also reviewed. We finally summarize the main future perspectives of MTSs as next-generation PSs within the context of cancer theranostics.

**Graphical Abstract:**

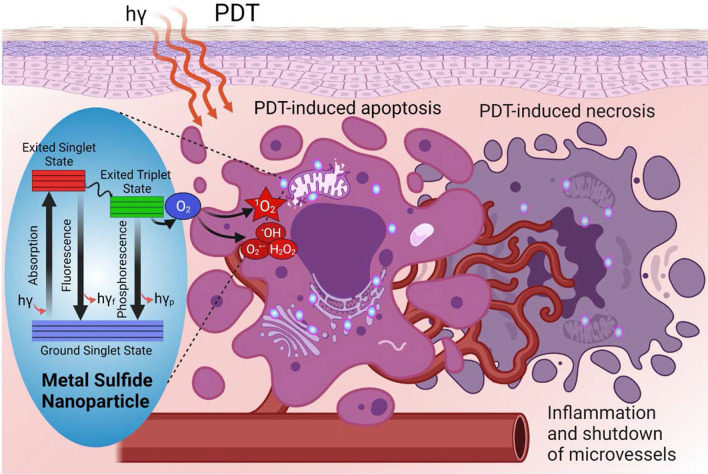

## Introduction

Cancer, a tumor or malignant growth arising from the division of abnormal cells, has caused an estimated 19.3 million new cancer cases (18.1 million excluding nonmelanoma skin cancer) and almost 10.0 million cancer deaths (9.9 million excluding nonmelanoma skin cancer) occurred in 2020, according to recent estimates of the worldwide mortality rate released by the International Agency for Research on Cancer [1]. The deaths are projected to rise to 28.4 million in 2040. The most diagnosed cancers are lung (2.2 million), breast (2.3 million), and colorectal (1.9 million) [1]. Facing this statistical catastrophe through regular prevention-focused approaches and early intervention programs falls short of patients’ expectations, who demand therapeutic alternatives to be affordable, side-effect discriminated, and more efficient and effective. Among the existing cancer treatments are surgery, chemotherapy, radiation therapy, targeted therapy, immunotherapy, laser treatment, stem cell transplant, hyperthermia, and small molecule-based therapy, being those of immunosuppressive-type most commonly used [2, 3]. However, although chemotherapy and ionizing radiation can destroy malignant growths at sufficient drug/radiation doses, they are noxious to the bone barrow and provoke immunostimulatory effects such as induction of heat-shock proteins [4]. In addition, cumulative radiation doses are restricted in radiation therapy, whereas systemic side-effects are still an issue in chemotherapy [5]. It has also been observed a substantial reduction of the natural killer cell function and diminution of lymphocytes in surgical resection of tumors, which are accompanied by high recurrence rates [6, 7] Thus, the desired cancer therapy must surmount these hurdles, and not only annihilate the main tumors but also recognize, localize and destroy any surrounding cancerous cells (even at distant micrometastases) by simultaneously setting off the immune system [8, 9].

Photodynamic therapy (PDT) is a versatile oncologic therapeutic modality and has been widely used clinically due to its intrinsic minimal noninvasiveness and high selectivity that enable to minimize the adverse impact on healthy tissues [10–13]. PDT employs three major components—light, photosensitizer (PS), and molecular oxygen. The visible light excites the PS accumulated in cancer cells, and the excited PS transfers photon energy to surrounding molecular oxygen to produce reactive oxygen species (ROS) that leads to death of cancer cells [12, 14, 15]. The PDT offers excellent repeatability without cumulative toxicity, low long-term morbidity, desired functional and cosmetic outcomes, and enhanced quality of life of patients [7]. The therapeutic efficacy of PDT greatly relies on properties of the PS such as aqueous solubility, greater tumor selectivity, high chemical purity, strong photosensitivity, and light absorption rate [11]. The idealization of PDT dates back to the early beginning of twentieth century, when a German medical student, Oskar Raab, serendipitously found that infusoria and species of *Paramecium caudatum* could be killed, when exposed to sunlight in the presence of a cell-specific dye called acridine [16, 17]. This was the first report on how PDT uses PS and since then, several varieties of synthetic (aniline dyes, eosin, fluorescein, etc.) and natural (*L. racemose*, Rose Bengal, *A. procera*, etc.) drugs have been developed as PSs [12]. To date, more than thousands of PSs, including natural and synthetic, are reported [18–20].

The first-generation PSs, such as hematoporphyrin derivative (HpD) and photofrin, and second-generation PSs, such as benzoporphyrins, purpurins, texaphyrins, and protoporphyrin IX (PpIX), have received extensive clinical uses [21]. However, most of these PSs possess complex composition and have low light absorption rate. Further, poor water solubility of these PSs precipitates aggregation, leading to quenching effect [21]. Currently, there are active research in progress in the field of nanotechnology in developing PSs with greater tumor selectivity, enhanced hydrophilicity, and strong photosensitivity to yield a reliable photodynamic reaction. The studies show that metal nanoparticles such as gold, silver and copper, manganese, aluminum nanoparticles; organic–inorganic hybrid nanoparticles, metal oxide nanoparticles, metal sulfide nanoparticles, Cd-free nanostructured metal chalcogenides (NMCs) have been extensively investigated in PDT [22–25]. While nanoparticles surface endows increased chemical activity, they require relatively low-power radiation to lead photo-stimulated reaction to generate singlet oxygen [26].

In this review, we discuss the recent development of Cd-free NMCs as alternative PSs and focus on the role of metal sulfide nanostructures in PDT for treatment of cancer. We consider metal sulfide nanomaterials for ROS generation to advance the photodynamic method. The recent developments in terms of high-energy transfer efficiency, rational designs, and potential applications of Cd-free NMCs in cancer-targeted PDT will be also discussed. We discuss the self-aggregation of NMCs in passive tumor cell targeting. The treatment of deep-seated tumors by using these PSs upon preferential uptake by tumor tissues due to the enhanced permeability and retention (EPR) effect, is also reviewed. We finally summarize the main future perspectives of NMCs as next-generation PSs within the context of cancer theranostics.

## Fundamentals of Photodynamic Therapy

**Fig. 1:**
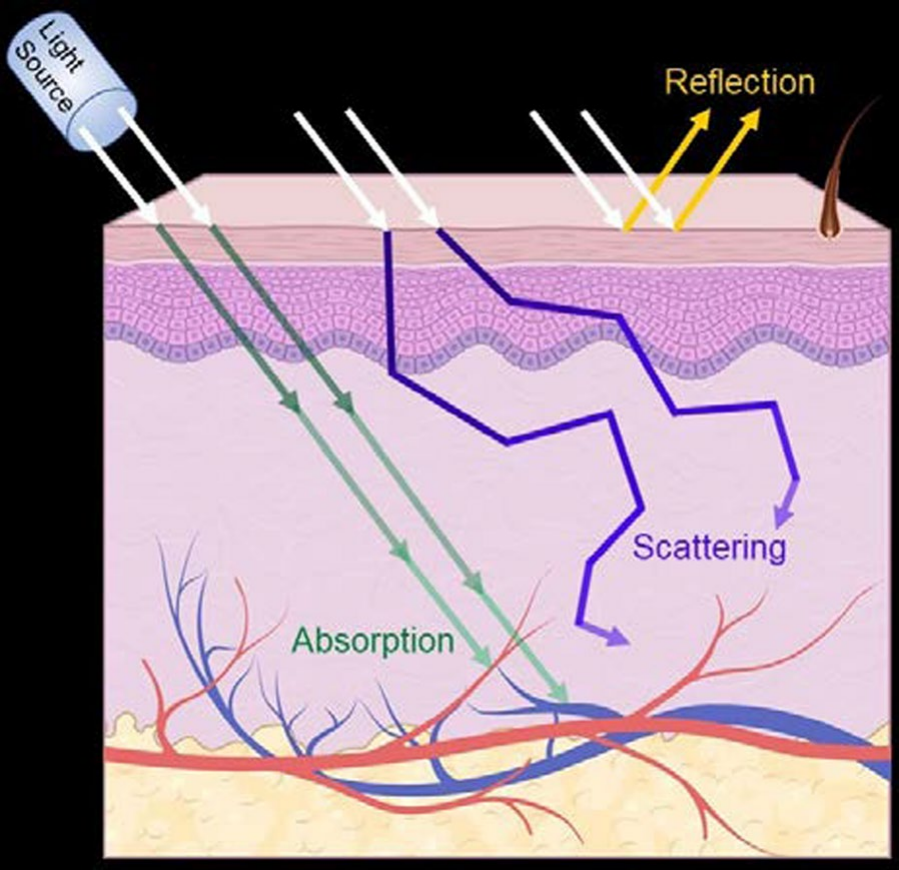
Propagation of light in biological tissue. Interaction of light with tissue results in reflection, absorption, and scattering

PDT includes the administration of a tumor-targeting PS drug followed by irradiation with visible light of particular wavelength corresponding to the absorption spectrum of the PS [20]. The energy of the excited PS is then transferred to molecular oxygen (^3^O_2_), which produces acutely-toxic singlet oxygen (^1^O_2_), a specific ROS, and superoxides. ^1^O_2_ is crucial for inducing tumor cell ablation via oxidation of key cellular macromolecules [7, 27]. A depiction that summarizes the interaction of light with tissue that results in reflection, absorption, and scattering is shown in Fig. 1. Selectivity in PDT depends on the degree of accumulation of the PS in the cancerous tissue and its vasculature, and on the restricted application of the incident light to the lesion zone. Out of the non-irradiated areas, the toxicity is minimal and can arise from the tissue tropism, due to daylight exposure or topical administration, that the PS shows [27, 28]. To date, the most widely used PS is photofrin, which is made up of purified aqueous mixtures of porphyrins [11]. This commercial PS has proven to be effective in certain neoplastic conditions, such as micro-invasive endo-bronchial non-small cell lung cancers, inoperable esophageal tumors, and neck and head malignancies [27, 29]. It has also been used in treating other types of cancer, including skin, superficial bladder, ovarian, breast, colon, and prostate [13, 30–32].

### Photophysical and Photochemical Processes

PDT is an intrinsic, dynamic process of the interaction of light, PS, and oxygen. A proper coordination of the light source, light delivery and the PS is crucial for an effective therapeutic delivery in PDT [33]. The dynamic interaction processes can be categorized into (i) photophysical process—light–tissue interaction and light–PS interaction, and (ii) photochemical process [34]. The incident light interacts with optically inhomogeneous biological tissues upon the delivery of light, leading to attenuation of light energy due to reflection, scattering and absorption [35], as shown in Fig. 1.

The reflection and refraction (refraction is not shown in Fig. 1) occur at the interface between two media with mismatched refractive indices and are governed by Fresnel’s law and Snell’s law, respectively. The resulting loss of light intensity directly relates to the relative values of the refractive indices of the media. The intensity loss can be minimized by application of light in perpendicular direction [33]. Scattering of light in tissue is the most paramount interaction contributing to approximately 90–99% of the total light attenuation [36]. This results in dispersion of light (widening of light beam) and eventual loss of fluence rate, known as power per unit area of light in W m^−2^ [35]. While inelastic scattering (Raman scattering and Brillouin scattering) influences negligibly the PDT, elastic scattering such as Rayleigh scattering, and Mie scattering play a dominant role. The Rayleigh scattering is a more wavelength-dependent process and occurs when the size of atoms or molecules in tissue is much smaller than the light wavelength (*λ*/10 > particle size, where *λ* is the light wavelength). In contrast, the Mei scattering is a non-wavelength selective process and occurs when the interacting molecules are comparable or larger than the light wavelength [37].

The absorption of light in tissue occurs when the photon frequency matches the frequency associated with the molecule’s transition energy and is influenced by the optical properties, size, shape, and density of the tissue elements [38]. Water, hemoglobin, melanin, cytochromes, elastin, and collagen are the major highly absorbing molecules in tissue. Together, absorption and scattering cause light attenuation resulting in loss of light intensity and leads to reduced scattering coefficient (${\mu }_{S})$ and absorption coefficient (${\mu }_{a})$ [33]. The light intensity (${I}_{x}$) at given depth “*x*” in the tissue can be determined as ${I}_{x}= {I}_{0}{e}^{-({\mu }_{a}+ {\mu }_{S})x}$, where ${I}_{0}$ is the initial light intensity [34]. The effective penetration depth in PDT for solid tumors is defined as the depth “x” where ${I}_{x}$ decreases to 37% of ${I}_{0}$. Most of the clinically used PSs show a Soret band at approximately 400 nm and multiple absorption peaks between 600 and 700 nm (Q-bands), which yields light penetration depth of approximately 3–5 mm depending on the tissue. Therefore, the use of PSs with absorption peaks at wavelength 700 nm or longer is preferred for deeper penetration of the light in tissue [38].

While a PS molecule in its ground singlet state (S_0_) consists of paired electrons with total spin (S = 0) and multiplicity = 1, its exited singlet states (S_*x*_, where *x* = 1, 2, 3, …with increasing energy state) are subdivided into multiple vibrational levels. The absorption of light by PS results in transfer of electrons to the short-lived (nanoseconds) exited singlet state. The electrons in the exited singlet state, eventually, fall to S_0_ upon relaxation. According to the vibrational relaxation (VR), an electron in a higher vibrational level of an exited singlet state (e.g., S_1_) promptly falls to lowest vibrational level of the excited singlet state (S_1_), dissipating heat energy. Following VR, the electron ultimately falls back to S_0_ via fluorescence emission or release of heat. The emitted light possesses higher wavelength (or lower energy) than that of originally absorbed light. Also, the electrons from S_1_ may be caught into long-lived (milliseconds) triplet excited state (T_1_) in between the exited and ground singlet states due to a non-radiative process known as intersystem crossing, where the electrons are no longer paired with the ground state and possess parallel spin. A rapid and stepwise VR within the vibrational levels in T_1_ can lead the electrons back to S_0_, emitting phosphorescence [10, 34].

**Fig. 2:**
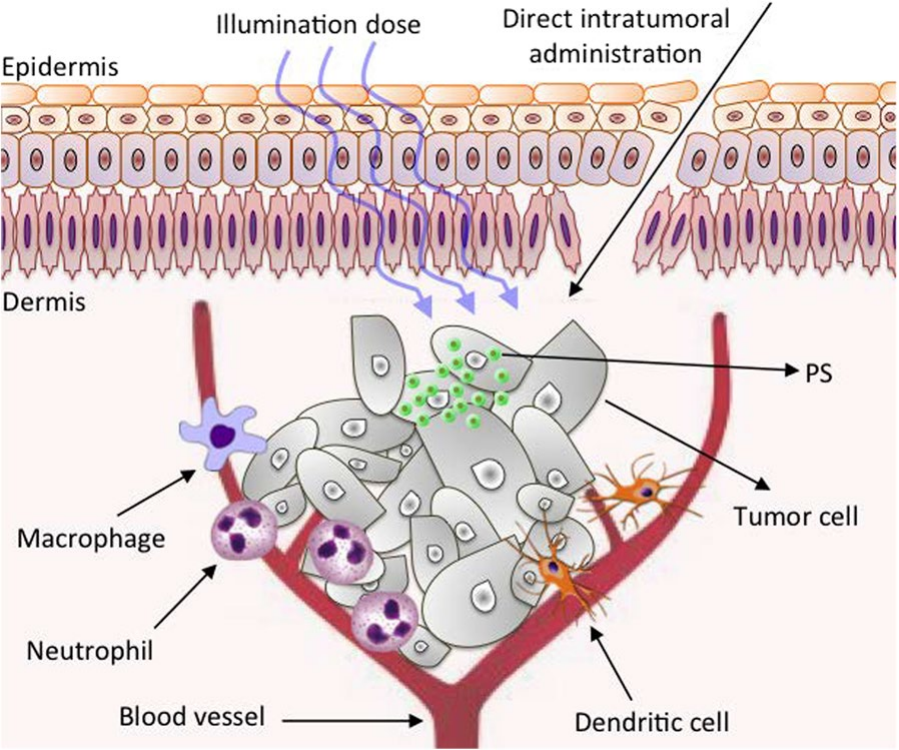
Fundamental photodynamic therapy mechanism to treat shallow/deep tumors via intratumoral injection. Significantly modified from Ref. [9]

**Fig. 3:**
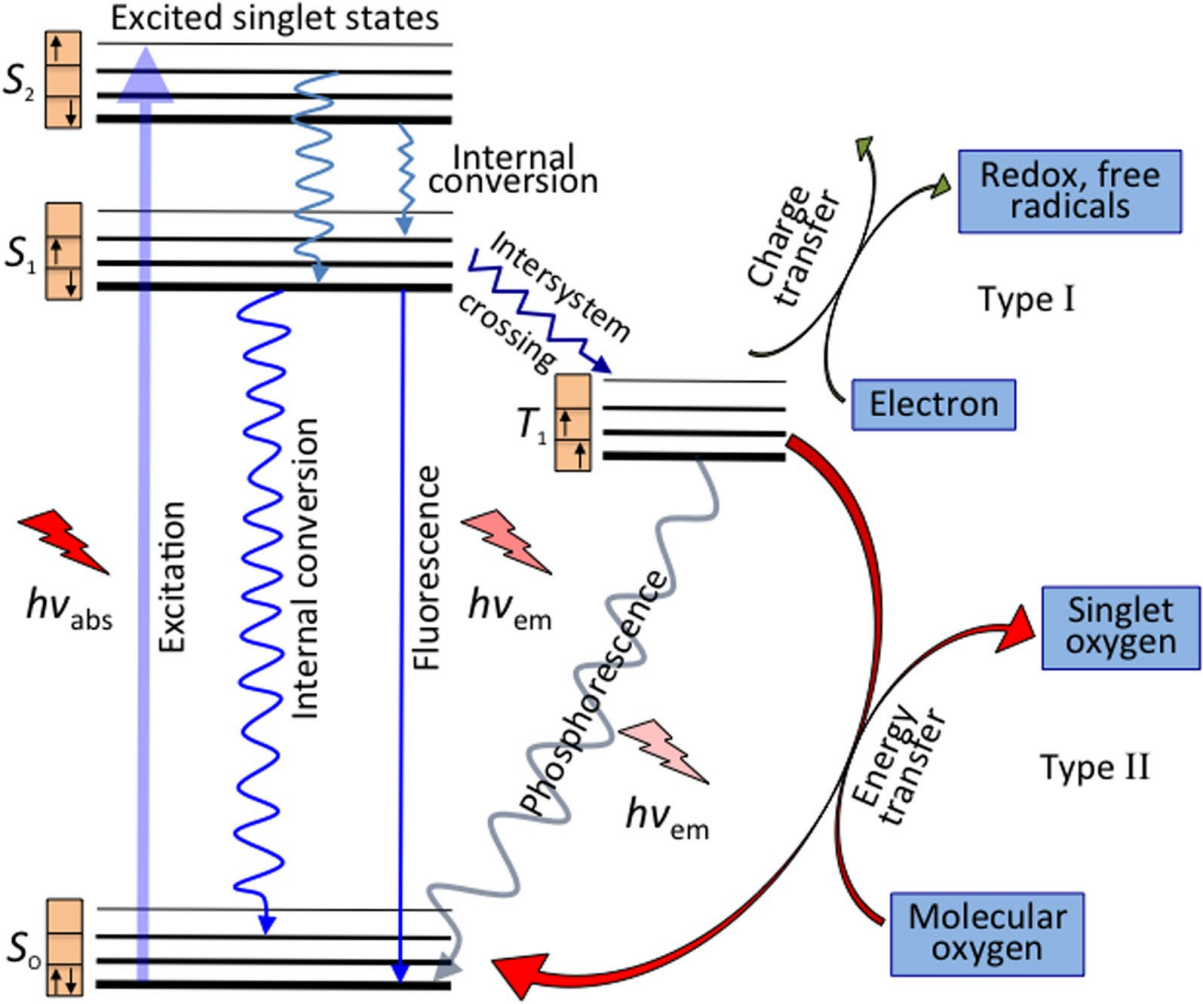
Generation of reactive oxygen species and excited states of the photosensitizer. Light promotes the excitation of an electron from a low-energy singlet state (S_o_) to high-energy singlet states (S_1,2_). Such states can lose their energy via fluorescence (radiative emission, light) or internal conversion (non-radiative emission, heat). The spin flipping of the high-energy electron takes places via intersystem crossing, which leads to a long-lived excited triplet state (T_1_). Type I and II reactions favor the formation of free radicals and singlet oxygen (^1^O_2_), respectively, in the presence of ^3^O_2_. Significantly modified from Ref. [11]

The excited PS then interacts directly with either surrounding ^3^O_2_ (triplet ground state) to generate ^1^O_2_ via energy transfer (~ 950 meV, type II), or any substrate to form free radicals via charge transfer (proton/electron, type I). The fundamental photodynamic therapy mechanism via intratumoral injection and the generation of reactive oxygen species and excited states are displayed in Figs. 2 and 3. In type I reaction, the free radicals may further react with oxygen to produce ROS. Also, both reactions may take place simultaneously being their kinetics strongly favored by the oxygen, substrate concentration, and type of PS. Superoxide anion initially produced via type I pathway by monovalent reduction does not cause oxidative damage but reacts with itself to generate oxygen and hydrogen peroxide (H_2_O_2_), which is catalyzed by the enzyme superoxide dismutase [39]. Superoxide also promotes the donation of one electron to reduce metal ions, which act as catalyst to convert H_2_O_2_ into hydroxyl radical ·OH. Both H_2_O_2_ and ·OH are important in biological settings since most cells have metals, which facilitate such catalyst-based reaction, and they can easily pass through cell membranes and cannot be excluded from cells [8]. Highly reactive HO^−^ induces not only damage through rate-limited diffusion, but also add new radicals that react with other molecules in a chain reaction, which together with ^1^O_2_ (produced by type II pathway) causes oxidative damage in lipids, fatty acids and DNA in areas proximal to the PS localization.

### PDT Mechanism for Tumor Destruction

The PDT-induced tumor destruction primarily occurs both in apoptotic (programmed cell death) and necrotic (non-programmed cell death) pathways [10, 29]. The light-activated PS can exert its tumor destruction effect either through direct tumor cell killing (occlusion of the tumor-associated vasculature) or modulation of the immune system [26]. Regarding the first mechanism, ROS species generated by the photoexcitation of PSs cause the oxidation of the lipids, fatty acids, and DNA in areas proximal to the PS localization. This oxidation in turns induces irreversible damages into the cellular compartments (i.e., mitochondria, lysosomes, membrane) that lead*s* to cell apoptosis, necrosis, or autophagy. These three mechanisms depend on a variety of parameters, including the nature of the PS applied, the PDT dose, as well as the genotype of the cells. For instance, high intensity light doses generally trigger necrosis, while low intensity trigger apoptosis [10]. On the other hand, if the PS is localized in the intracellular compartments the cell death will probably occur through an apoptotic pathway, while if the PS is localized in the plasma membrane or lysosomes, the cell will probably undergo a necrotic or an autophagic pathway.

The localization of the PS is also controlled by its surface properties. Hydrophobic compounds have been found to rapidly diffuse into the tumor cells and localized in the intracellular compartments compared to more polar compounds, which generally internalized through active processes that are slower and tend to localize on the outer cellular compartments. All these controllable factors allow to develop more personalized and efficient PDT treatments. The second proposed mechanism relies on the damage of the tumor vascular system. Similar to tumor cells, the vessels are abnormal and have poor and incomplete cellular borders [40] that facilitates the PS accumulation, which after activation causes a disruption in the vascular walls and cells, inhibiting the blood flow to the tumor decreasing the oxygen levels intake by the tumor (hypoxia) [41]. This event leads to necrosis of both tumor cells and vasculature, whose lysis stimulates the release of a high amount of intracellular debris that further blockage and collapse the micro vasculature feeding of the tumor [10, 42]. The PDT-treated dying cells (tumor cells and vasculature) also release different inflammatory mediators such as proteinases, peroxidases, cytokines, growth factors, and other inmunoregulators [9, 43], that activate an immune response (third mechanism). The initiation of this immune cascade attracts various immune cells, such as macrophages and neutrophils to the treatment region that further contribute to the tumor destruction by phagocytosing PDT damaged cancer cells and activating cytotoxic T cells and dendritic cells that induce necrosis or apoptosis whenever tumor cells are found [43, 44]. Although all these mechanisms combined or separated induce mechanistic cell toxicity, a more controlled cell killing process can be achieved by tailoring the properties of PSs.

### Properties for Ideal Photosensitizers

PSs play a critical role in PDT to dictate how efficiently ^1^O_2_ is generated. The PDT-induced anti-tumor effects and the treatment efficacy of PDT mainly rely on the properties of PSs. To enhance treatment efficacies, an ideal PS should possess the following properties: (1) high tumor selectivity and subcellular targeting capability; (2) strong absorption with high extinction coefficient at near-infrared (NIR) wavelength range (700–1300 nm), where tissue penetration is maximized and the auto-absorption is minimized by other endogenous molecules (including hemoglobin); (3) negligible dark toxicity; (4) high quantum yield of ^1^O_2_ generation; (5) inexpensive and economically feasible; and (6) excellent aqueous solubility. As the development of novel PSs has been evolving, many of these properties were successfully attained which were used to sort them out. The first-generation PSs were developed in the 1970 that included porphyrin-based PSs [13]. These PSs were highly effective in first clinical trials, showing efficient tumor destruction, negligible dark toxicity and easy formulation in water-soluble preparations [7]. However, prolonged patient photosensitivity (poor clearance), low absorption of light (*e* = 1170 M^−1^ cm^−1^), and sub-optimal tumor selectivity have limited its use in PDT. In order to overcome these limitations, a second generation of PSs were developed which included porphyrin derivatives, phthalocyanines, chlorins, anthraquinones, curcuminoids and its metalated derivatives (i.e., aluminum phthalocyanine tetrasulfonate and Si(IV)-naphthalocyanine). These PSs presented higher quantum yields of ^1^O_2_, absorption at longer wavelengths (630–850 nm, providing deeper tissue penetration), shorter tissue accumulation, and cutaneous photosensitivity. Nevertheless, their poor tumor accumulation, aggregation, and self-quenching in aqueous solutions (as a result of their hydrophobicity) diminish their PDT efficacy. To tackle these drawbacks, PSs have been conjugated to biological targeting molecules, such antibodies, and peptides, or encapsulated in other carriers (e.g., polymers, micelles, and nanoparticles) leading to the third generation of PSs. This approach significantly increased the tumor selectivity and enables to minimize: (i) the damage of surrounding healthy tissues, (ii) side effects including prolonged skin photosensitivity, and (iii) invasiveness [11].

## Nanoparticles in PDT

Recently, the use of nanoparticles (NPs) has been proposed as carriers of PSs due to their unique properties, such as (1) synthetic feasibility; (2) ease of functionalization with target moieties that increase PSs biodistribution, pharmacokinetics, cell uptake, and selectivity; (3) ability to transport hydrophobic drugs intravenously; (4) large volume distribution increasing high delivery of PSs into target sites; (5) easier internalization and retention into tumor tissues via the EPR effect given the tumor leaky vasculature; (6) controlled release of drugs; (7) protection of PSs against degradation and prolonged circulation in the bloodstream; and (8) versatility to incorporate other existing therapies or diagnosis modalities to PDT [7, 27, 45–47]. The use of NPs as cargo for PSs has been broadly divided into two categories: Biodegradable (natural or synthetic polymer-based NPs) and non-biodegradable (ceramic or metal-based NPs). Likewise, given that the distinct nanoformulations dictate the active intermediary role of NPs in the process of photodynamic activation, a new classification was proposed by Chatterjee et al., namely, as passive, and active nanocarriers [45]. In the latter, NPs participate in photodynamic process either as energy transducers or PSs themselves. In the former, nanostructures absorb the incident light at wavelengths transparent to the body and then transfer it to the PSs, thus allowing the possibility to treat deep-seated tumors. Some of the NPs used for this approach include up-conversion NPs, noble metal NPs, metal sulfides/oxides, and carbon-based nanomaterials, which have shown to be relevant for in vitro and in vivo studies. Nevertheless, some major limitations of existing organic PSs (such as low extinction coefficients and poor photo-stability) pose a big challenge for clinical applications [11, 26].

In this sense, the innovation of inorganic nanostructures with the intrinsic ability to produce ROS upon the absorption of light has open new arenas for their use as PSs. Some of the first-evaluated inorganic NPs included TiO_2_, ZnO, fullerene, and Cd-based quantum dots [48, 49]. Although these PSs compared to conventional organic PSs exhibit several advantages, some issues ascribed to low absorption at longer wavelengths have limited their translation to clinical applications. Recently, NIR-absorbing metal NPs (e.g., Au, Pd) have been evaluated as PSs due to its high biocompatibility and tunable surface plasmon resonance absorbance, which not only induces ROS production but can also generate light-to-heat conversion (photothermal effect). Their preparation, however, requires tedious and expensive synthetic procedures that increase their cost effectiveness. Moreover, their non-biodegradability raises toxicity concerns which further limits their clinical applications. Over the past few years, other metal NPs have been evaluated, e.g., NIR-absorbing copper sulfide (CuS) NPs due to its low cost and biodegradability. Previous reports indicate that these NPs possess low cytotoxicity, high photostability and photothermal conversion efficiency, intriguing photodynamic activity, tunable light absorption to longer wavelengths, higher molar extinction coefficients (at least 3–7 orders of magnitude), resistance to enzymatic degradation, excellent water dispersibility, superior ability to be conjugated to various biomolecules, and versatile to be incorporated into relevant multifunctional theranostic systems [50, 51]. More in general, the photodynamic therapeutic effect of nanostructured metal sulfides either as next-generation PSs is being currently explored for cancer treatment or as photothermal agents to treat other diseases, which will be discussed in detail in the next section.

## Metal Sulfides as Photosensitizers in PDT

Among the most studied NMCs in PDT are the metal transition sulfides (MTSs), with a general formula MS_2−*x*_, which exhibit unique physical and chemical properties. Depending upon their metal composition, it can crystallize in three main different structures: (i) molybdenite type (MoS_2_), (ii) CdI_2_-type, and (iii) pyrite (FeS_2_). This variability in composition and structure is responsible for MTS nanostructures to have innate physicochemical merits, which have been applied in light emitting devices, photovoltaic devices, catalysts, sensors, and more recently, in theranostics [52]. As for theranostic applications, they have promising advantages to be used as bio-imaging probes, drug delivery cargos, and phototherapy agents due primarily to their strong absorption in the NIR region (around 700–1100 nm), high extinction coefficients, versatile surface chemistry, high fluorescence, magnetism, structural, and thermal stability, which has boomed significant research in the field of MTSs. Moreover, MTSs are easily obtained through low-cost synthetic methods compared to other NIR absorbing metal NPs, such as gold, silver, and copper [52]. For instance, the cost of 1 mol of Au atoms is estimated to be around $52,200, while the same amount of 1 mol of CuS molecules costs nearly $330 [50]. We highlight below the use of the main types of MTSs as passive and active photosensitizer agents recently published in the literature.

### Molybdenum Disulfide

Molybdenum is a transition metal with electronic configuration [Kr] 4*d*^5^ 5*s*^1^ and exhibits superior catalytic reactivity because of its half-filled *d* and *s* orbitals. Molybdenum disulfide (MoS_2_) part of the transition metal dichalcogenide families are generally prepared by incorporation of Mo sheets into sulfur sheets, which are kept together by intense covalent interactions. MoS_2_ nanosheets are atomically‐thin two-dimensional (2D) layers analog to graphene that have shown great potential in a wide range of fields, including biomedicine given their unique optical, electronic, and mechanical properties. Reports show that nanostructured MoS_2_ could interpenetrate cells via endosomal cell uptake and pinocytosis [53]. In terms of NIR absorbance, Chou et al*.* first demonstrated that single-layer MoS_2_ sheets have higher absorbance in the NIR region than graphene and better extinction coefficient compared to gold nanorods. This study substantially prompted extensive research thereof as NIR-triggered drug delivery platforms and photothermal agents [54]. In PDT, MoS_2_ nanosheets have been mostly used as a passive platform due to their extraordinary surface‐area‐to‐mass ratio 2D structure that enables them to load therapeutic molecules more efficiently. In addition, their innate photothermal properties endow these therapeutic platforms with intriguing synergistic effects suitable for cancer treatment. Liu and co-workers were the first to report one of these platforms that consist of lipoic acid-terminated polyethylene glycol (LA-PEG) MoS_2_ nanosheets loaded with PS chlorin e6 (Ce6) [55]. They observed a higher cellular uptake of Ce6 upon light irradiation of 800 nm and a significant enhancement in PDT efficiency *in vitro*. This enhancement was ascribed to a mild hyperthermia effect that increases membrane permeability and in turn promotes higher cellular uptake. The photothermally enhanced PDT synergistic effect was similarly demonstrated *in vivo* inducing controlled delays in 4T1 tumor growth in injected mice. A comparable system was reported by Jia and co-workers working with Ce6 labeled with an ATP aptamer before being loaded to MoS_2_ nanoplates, which produced a desired drug delivery response [56]. Their studies indicate that after nanoprobe internalization ATP-abundant lysosomes induced the release of the single-stranded aptamer from MoS_2_. Subsequently, the Ce6 fluorescence (excitation wavelength 633 nm) allowed the imaging of intracellular ATP and generation of ^1^O_2_. This turn-off nanoprobe system reduced the dark toxicity associated to Ce6 and showed enhanced anticancer properties.

**Fig. 4:**
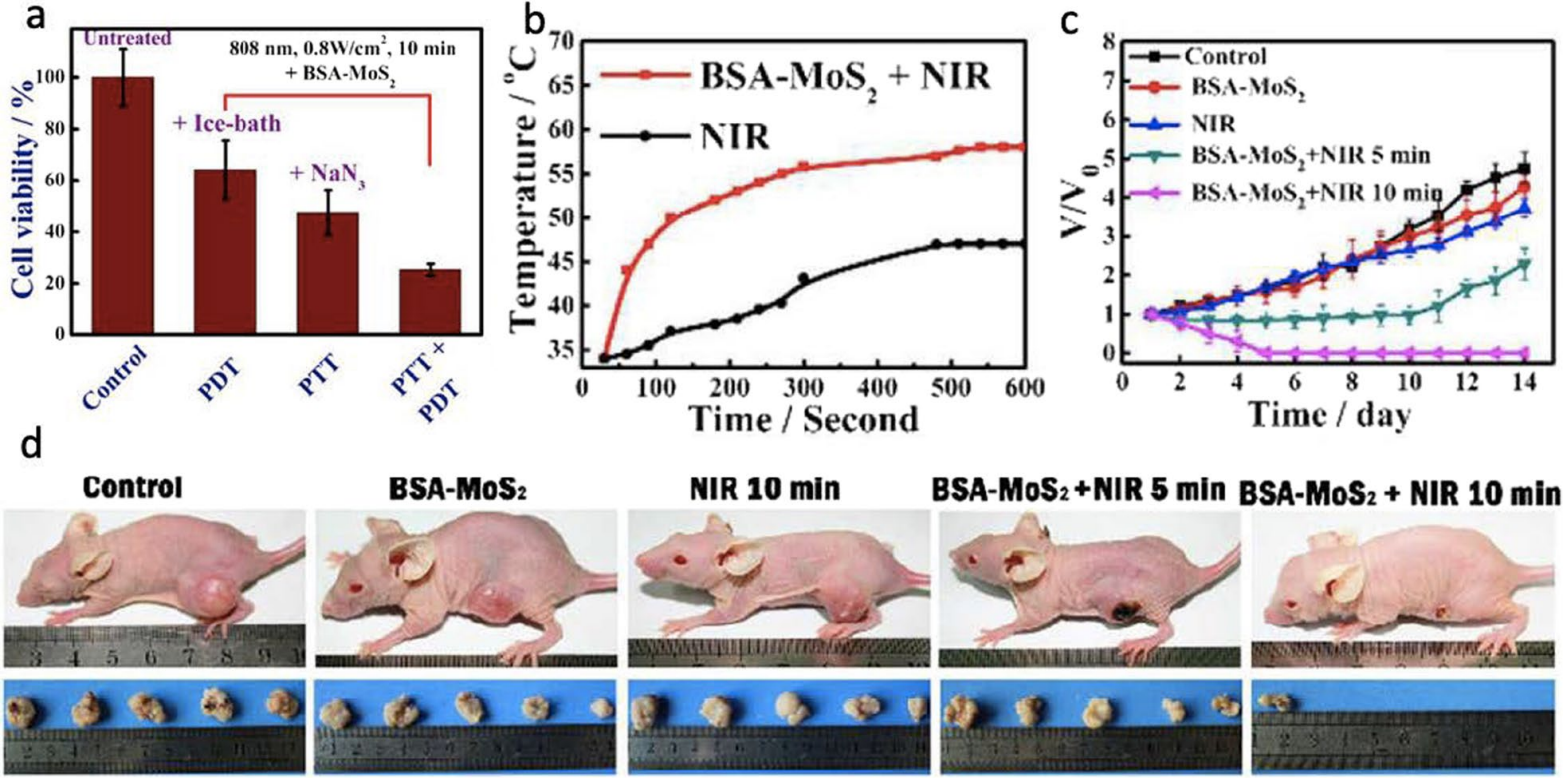
Relative cell viability of HepG2 exposed to with BSA-MonS_2_ after different treatments, including untreated group, only PDT group, only PTT group and PD/PT combined group. **b** Temperature profiles of tumor-bearing m**a**ice under NIR irradiation (with PBS or BSA-MoS2 injection); **c** Relative tumor volume of mice. **d** Representative photographs of mice and tumors. Reproduced with permission from Ref. [61]. Copyright 2017, Royal Society of Chemistry, Washington, DC

Nanostructured MoS_2_ has been also regarded as a potent alternative to graphene due to its 2D structural similarity, excellent charge-density wave transition, conductivity, biocompatibility, and superb electrical properties. Recently, for instance, Liu et al. have shown that nanostructured MoS_2_ was less hepatotoxic in comparison with graphene oxide [57]. Compared to graphene oxide, nanostructured MoS_2_ has a wide range of appropriate properties for applications in energy storage, catalysts, and biomedicines because of their low-cost, exotic features, and physicochemical characteristics [58–60]. Specifically, the great biocompatibility, extremely effective valency, and high sensitivity of nanostructured MoS_2_ are more promising than graphene oxide to expand and design nanoprobes for optical imaging, drug delivery, medical bioimaging, and foremost for cancer phototherapy [54, 56, 61]. A “four-in-one” nanoplatform, for instance, based on bioconjugated MoS_2_ was constructed and designed by Song et al. to discover favorable imaging-guided phototherapy, photothermal therapy (PTT), and PDT, where the nanostructures were synthesized via the hydrothermal method [61]. The group proposed that nanostructured MoS_2_ can be used as a PS material for PDT in cancer treatment (see Fig. 4). In this study, the authors intermixed bovine serum albumin (BSA) with MoS_2_ to modify biocompatibility responses and then conjugated with Cy5.5 (a bright, near-IR fluorescent dye) to obtain innovative fluorescence imaging features, as shown in Fig. 4. Through in vitro and in vivo experiments, the nanoplatform was proved to induce photoablation of tumor cells and tissues (see Fig. 4). B-ultrasonography and MR imaging were used to monitor the solid tumor removal after therapy, which showed a liquefaction necrosis process for rehabilitation.

In a separate account, a multifunctional PC10A/DOX/MoS_2_ hydrogel system was prepared by Jin *et*.*al* for chemotherapy/PTT/PDT of 4T1 tumors [62]. The authors also investigated the immune responses caused by the photothermal and photodynamic effect of MoS_2_ nanosheet in the hydrogel. PC10A/DOX/MoS_2_ were produced by loading positively charged DOX (drug) and negatively charged PC10A (hydrogel) on the surface of MoS_2_ nanosheets through a layer-by-layer approach, which resulted to be injectable and possess great biocompatibility. Two-dimensional MoS_2_ nanosheets in the hydrogel were then tested as photodynamic agents controlling ROS production. The results indicate that PC10A/DOX/MoS_2_ hydrogel in presence of laser exposure can have antitumor immune influences to prevent the development of primal 4T1 breast tumors and distal lung metastatic nodules. The overall findings proved that PC10A/DOX/MoS_2_ hydrogel is beneficial for antitumor immunity therapy to treat malignant tumors via PDT. Similarly, Xu et al. (2017) IR-808 dye sensitized upconversion nanoparticles (UCNPs) with a chlorin e6 (Ce6)-functionalized silica layer, which was then integrated with MoS_2_ nanosheets for imaging-guided PDT [63]. In this study, MoS_2_ nanosheets were synthesized through liquid exfoliation of bulk MoS_2_ flakes, which were prepared by molten salt electrolysis method. The nanoplatform shows both abundant ROS and local hyperthermia when exposed to a single 808 nm laser irradiation on the photosensitizer. These in vitro and in vivo tests suggest that this nanoplatform offers great cell killing and tumor inhibition efficacy when both PTT and PDT approaches are combined.

It is noteworthy that some Cd-free NMCs (e.g., molybdenum diselenide, MoSe_2_) can be also considered as innovative PTT agents with therapy efficacies comparable to MoS_2_. MoSe_2_ exhibits a direct band gap of ∼ 1.5 eV (compared with MoS_2_ ∼ 1.8 eV) and excellent long-wavelength NIR absorption, which can induce stronger penetrability in deep-tissue photothermal therapy [64]. A nanosystem of indocyanine green (ICG)-loaded MoSe_2_ nanoparticles (MoSe_2_@ICG-PDA-HA) with dual photothermal/photodynamic functions under near-infrared irradiation was developed by Liu et al. [64]. They reported that MoSe_2_@ICG-PDA-HA can contribute to the production of ROS after a minute exposure of 4T1 tumor cells without affecting D-α-tocopherol succinate-treated groups with free ICG or prior MoSe_2_ NPs. The authors further found that ICG and MoSe_2_@ICG-PDA-HA could produce ROS during irradiating with 808 nm NIR laser, which decreased the extra anti-ROS factor (tocopherol). To explore the inhibitory effect of MoSe_2_@ICG-PDA-HA, the authors incubated the multicellular spheres with PBS, ICG, MoSe_2_ NPs, and MoSe_2_@ICG-PDA-HA about 24 h, respectively, and then irradiated 5 min by laser (0.5 W cm^−2^). The MoSe_2_@ICG-PDA-HA + Laser group showed the best inhibitory effect on multicellular spheres growth, and the cell sphere volume became the original 41.8%, which was better than the MoSe2 NPs + Laser group (66.7%) and the ICG + Laser group (62.2%). This nanoplatform is a promising system that could enhance the photothermal/photodynamic synergy effect to effectively treat cancer.

### Zinc Sulfide

Another type of MTSs used to increase the efficiency of PSs in PDT is zinc sulfide (ZnS), which is a II–VI semiconductor compound that has various applications in nanomedicine [65]. Nanocomposite of reduced graphene oxide (rGO) and CuInS_2_/ZnS nanocrystals (CuInS_2_/ZnS/liposome-rGO NCs) was developed to investigate both PDT and PTT. In vivo studies in mice bearing tumors induced by esophageal cancer Eca-109 cells indicate that CuInS_2_/ZnS/liposome-rGO markedly reduced the tumor size, while in vitro studies revealed a significant induction of apoptosis in human esophagus carcinoma cells upon irradiation of 671-nm laser. The findings show that the nanocomposites not only can produce ROS in the presence of light (671-nm laser), but also turn light into heat energy. In addition, ROS and heat produced by CuInS_2_/ZnS/liposome-rGO were superior to those produced by CuInS_2_/ZnS/liposome and rGO nanosheets alone [66].

To understand more the role and capability of ZnS to produce ROS, water-soluble Mn-doped ZnS quantum dots (ZnS:Mn QDs) were investigated by Diaz-Diestra et al. and used as potential PSs [67]. The authors selected the RB/DPBF pair system because the photo-oxygenation pathway can generate ^1^O_2_ via a Type-II reaction. In this sense, ZnS:Mn can generate excited singlet state oxygen by energy transfer. They found that the quantum yield of ^1^O_2_ is 0.62 in buffer and 0.54 in water in the presence of a chemical scavenger and a standard dye when ZnS:Mn QDs were used as PS (532 nm laser). The authors emphasized that dependency of the reaction on dissolved O_2_ showed that ^1^O_2_ is produced by the QDs during the photosensitization process and there is no oxidation of DPBF in the absence of light source. The findings were in agreement with a chemical trapping energy transfer mechanism and demonstrate the capability of ZnS:Mn QDs not only as PDT PSs but also as luminescent nanoprobes for cancer theranostics. In a similar study, Martynenko et al. demonstrated an enhanced PDT efficacy for the destruction of Ehrlich ascites carcinoma (EAC) cells using Cd-free ZnSe/ZnS quantum dots (QDs) and chlorin e6 complexes (water-soluble QD-Ce6 complex), when irradiated with 405 nm diode laser (power density of 40 mW cm^−2^) [68]. The enhanced PDT efficacy of the complex was ascribed to the synergistic effect of the QDs on the Ce6 intracomplex photoexcitation energy transfer and on the increased cellular uptake of Ce6. Further investigation shows that in absence of Ce6 using similar ZnS-based QDs photodynamic effects were induced, which were used on pancreatic cancer cells [69]. After treating human pancreatic SW1990 cancer cells with QDs and irradiating with 365 nm light, the authors detected Bcl-2 and caspase-3 via real-time PCR and protein immunoblotting. In addition, cell viability was remarkably less in the presence of exposure or with a longer incubation time and a superior light dose. Ultrastructural variation in SW1990 cells with organelle degeneration and chromatin condensation and aggregation in the vicinity of nucleus were also observed by the authors when the QDs where light exposed. The authors concluded that the QDs can be used as a promising PS to inhibit SW1990 cell proliferation through ROS generation and apoptotic protein expression regulation.

### Copper Sulfide

**Fig. 5:**
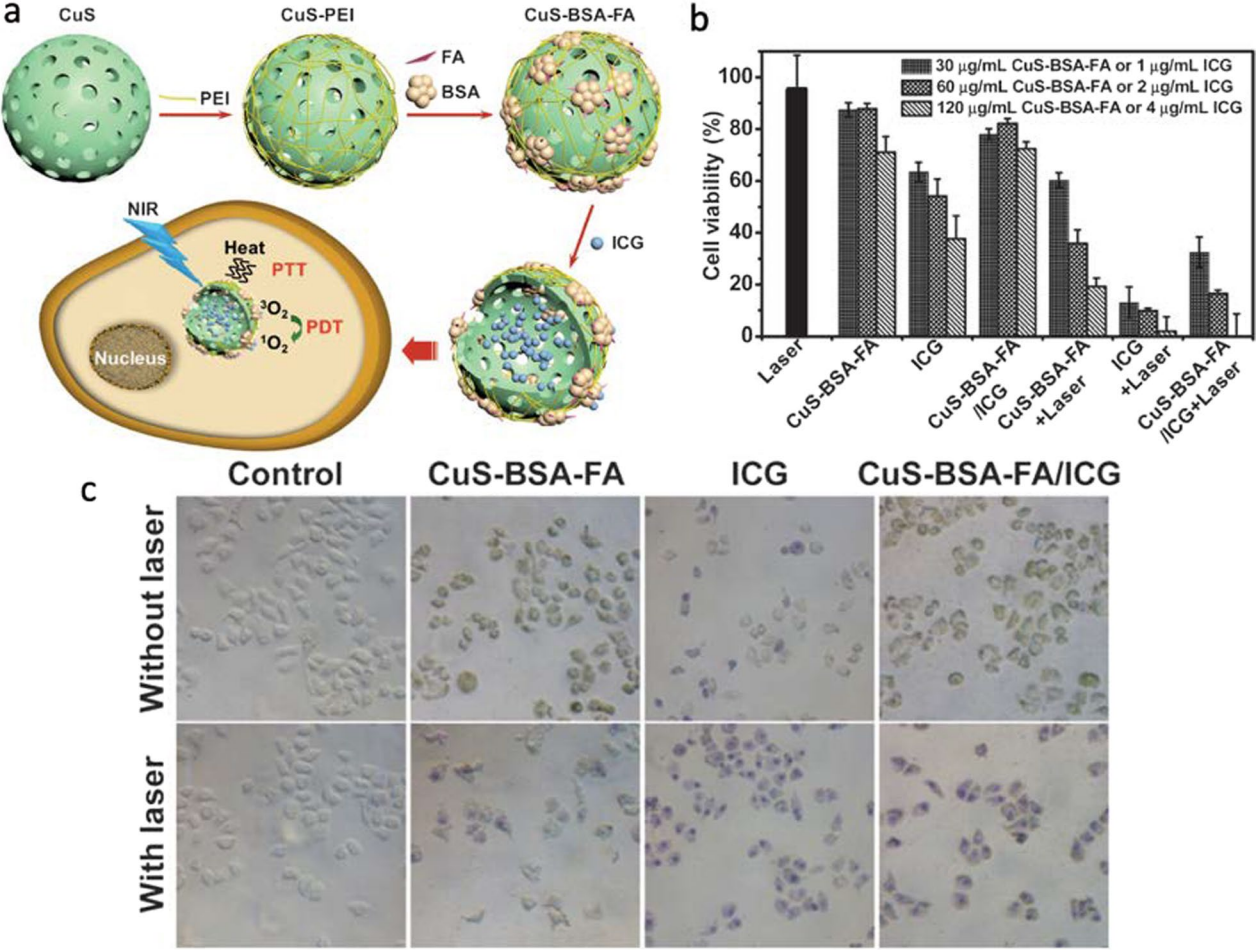
**a** Schematic illustration of the construction of CuS–BSA–FA as a vehicle to deliver ICG showing the mechanism of PDT and PTT therapies. **b** Cytotoxicity assays on HeLa cells in the presence of CuS–BSA–FA, ICG and CuS–BSA–FA/ICG with or without NIR irradiation (1 W cm^−2^ for 5 min). **c** HeLa cells in the presence of CuS–BSA–FA (60 mg mL^−1^), ICG (2 mg mL^−1^) and CuS–BSA–FA/ICG (60 mg mL^−1^ for CuS–BSA–FA and 2 mg mL^−1^ for ICG) with and without laser irradiation (1 W cm^−2^). Reproduced with permission from Ref.[51]. Copyright 2016, Royal Society of Chemistry, Washington, DC

Copper sulfide (Cu_2−*x*_S) NPs, a p-type semiconductor, have attracted increasing attention in recent years due to their excellent surface plasmonic absorbance in the NIR region, which can be exploited in theranostic applications. This property originates from the free holes of the unoccupied highest energy states of the valence band, which are strongly dependent on the crystal phase of the NPs and in turn on the Cu/S ratio [70]. Some of the most dominant structural phases include Cu_31_S_16_ (monocyclic phase), Cu_9_S_5_ and Cu_1.8_S (cubic phase), Cu_7_S_4_ and Cu_1.75_S (orthorhombic phase), Cu_58_S_32_ and Cu_1.81_S (triclinic phase), and CuS (hexagonal phase or covellite). It has been reported that more copper deficiency in the lattice structures (Cu_2−*x*_S with *x* > 0) is a result of the decrease in the Cu/S ratio, which increases the concentration of free carriers and induces the observed localized surface plasmon resonance (LSPR) absorbance in the NIR region [71, 72]. In a similar analysis, when the Cu/S ratio increases, as in Cu_2_S (with *x* = 0), the few free carriers (holes) decrease and LSPR is not observed. Besides varying compositions, tuning Cu_2−*x*_S NPs morphology can also alter their LSPR. However, through the current synthetic approaches, most of the reported morphologies fall within the microsize regime, hindering further biomedical applications [73]. Compared to plasmonic metals including gold NPs (whose free carrier concentration is fixed and consequently, their LSPR can only be tuned by varying the NP morphology and damping parameter), the LSPR of Cu_2−*x*_S NPs can be tuned throughout the NIR region upon varying composition and crystal structure [50, 51, 74]. Consequently, the cost of the therapy could be reduced given the abundance of this metal and the easily scalable synthesis of Cu_2−*x*_S, which makes them promising candidates as translational phototherapeutic agents. For instance, producing 1 mol of Cu_2−*x*_S costs around $330 and 1 mol of Au costs $52,200 [48]. Moreover, Cu_2−*x*_S is resistant to photobleaching and photodegradation and, due to its tailorable LSPR, it exhibits fine-tune absorption spectrum and large extinction coefficients in the NIR region. Han et al. [51] reported that bovine serum albumin (BSA)–folic acid (FA) functionalized hollow Cu_2–*x*_S nanostructures can be used as drug delivery vehicle to target indocyanine green (ICG, a NIR-absorbing phototherapeutic agent) to HeLa cells (see Fig. 5). It was demonstrated that this drug delivery strategy significantly improved the stability and reduced the dark toxicity of free ICG, which tends to aggregate in biological fluids. Moreover, the hybrid system exhibited a higher photothermal heating effect and capability to generate ^1^O_2_ under laser irradiation, when compared to bare nanocarriers. No obvious cell death was observed when treating the cells with CuS–BSA–FA and CuS–BSA–FA/ICG under dark conditions, however after NIR irradiation, cells were destroyed when CuS/ICG-NIR was used (see Fig. 5).

**Fig. 6:**
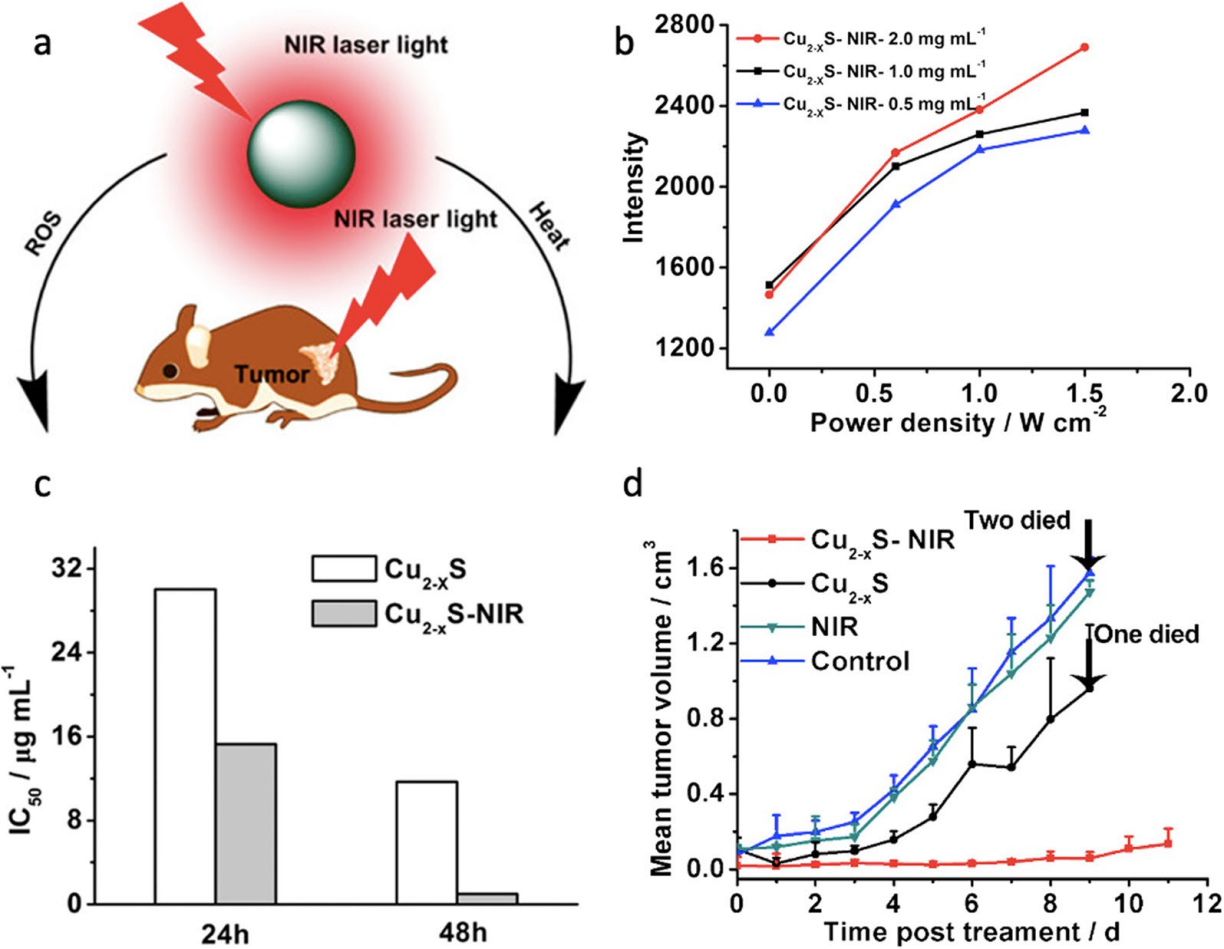
**a** Illustration showing the near-infrared photothermal and photodynamic therapeutic effect of plasmonic Cu_2–*x*_S NCs. **b** The intensity of ESR for the spin trapped hydroxyl radical produced by Cu_2–*x*_S NCs at various concentrations and light power densities. Viability of B16 cells at different concentrations of Cu_2–*x*_S NCs with and without NIR irradiation (808 nm, 2.3 W cm^–2^ for 3 min) measured after 24 and 48 h incubation. **c** Corresponding IC50 value of each group for 24 and 48 h. **d** Comparative efficacy study of single intratumoral injection in B16 xenograft bearing nude mouse models showing the mean tumor volumes for the Cu_2–*x*_S-NIR group. Reproduced with permission from Ref. [74]. Copyright 2015, American Chemical Society, Washington, DC

To date, the use of Cu_2−*x*_S as PSs in PDT has been significantly expanded. Wang et al. were the first to report that Cu_2−*x*_S NPs not only exhibited photothermal effect but also generated concomitantly high ROS levels under NIR laser light irradiation (at 808 nm and 0.6 W cm^−2^), which induced human melanoma B16 tumor destruction (see Fig. 6) [74]. Cu_2−*x*_S NPs were synthesized by a non-injection approach and capped with oleylamine. The average core diameter was 6.5 nm obtained from TEM analysis. To allow NP dispersion in polar solvents (including water and PBS at pH 7.4), oleylamine was exchanged with amphiphilic thiolated PEG molecules (carboxyl-PEG-SH, molecular weight 3 kDa, and methoxy-PEG-SH, 2 kDa). After the ligand exchange reaction, the authors noticed a blue-shift and intensity increase in the LSPR band ascribed to partial oxidation of the anion sublattice, which introduced additional holes in the upper edge of the valence band and consequently a shift in the LSPR to higher energies. The hydrodynamic size of Cu_2−*x*_S in PBS was approximately 12 nm, which could allow the NPs to easily extravasate and reach targeted tumor sites.

In the same study, the authors noted that an increase in power density (up to 2.3 W cm^−2^) resulted in a temperature increase (30.6 °C) of PEG–CuS solution within 10 min, yielding 16.3% of photothermal efficiency when assessed in PBS. A slight aggregation was observed at temperatures higher than 60 °C in PBS, which was attributed to the striping of PEG ligands without significant effects on the NPs photothermal efficiency. The photothermal conversion efficiency (PTCE) was also tested on human melanoma B16 tumor-bearing Balb/c nude mice. The NPs were intratumorally injected at a concentration of 15 (mg kg^−1^) in Cu, and the mice were irradiated with a NIR laser (at 808 nm, 0.6 W cm^−2^, and a spot size of 2 cm in diameter) for 100 s. Compared to the control group, the group of animals that received the NPs injection (also exposed to the laser treatment) showed a remarkable temperature increase by approximately 14 °C in the IR thermal map of the tumor region, which was enough to kill the tumor cells in xenografts (see Fig. 6). To shed more light upon the death mechanism pathway, the expression of HSP70, a biomarker for elevated stresses (i.e., elevated temperature), and ROS were measured. A high overexpression of HSP70 led the authors to further investigate the photodynamic properties of the NPs. The methods used to detect ROS were electron paramagnetic resonance (EPR) spectroscopy and a fluorescence assay. The former assay enables the detection of free radicals, such as ^•^OH and superoxide (^•^O_2_^−^). These radicals form an adduct composed of 5,5-dimethyl-1-pyrroline-N-oxide (DMPO) and 2,2,6,6-tetramethylpiperidine (TEMP) with a spin-trapping agent, which shows typical triplet and quadruple ESR signals. NP solutions at different concentrations (from 0.5 to 2 mg mL^−1^) were irradiated with NIR light irradiation (808 nm, 0.6 W cm^−2^ for 5 min) and the spectra were recorded. The resulting ESR spectra show the characteristic multiplicity 1:2:2:1 of DMPO-OH and a notorious enhancement of up to 83.5% in ^•^OH levels. No obvious ESR signal was detected for ^1^O_2_-spin trapped adducts. Also, the ROS generation induced by NIR light irradiation of Cu_2−*x*_S NCs was found to be both concentration and laser power dependent (Fig. 6). The second assay focused on ROS detection monitored the oxidation of non-fluorescent dye dichlorofluorescein diacetate (DCFHDA), which after exposure to ROS was converted to 2,7-dichlorofluorescein (DCF, a highly fluorescent derivative at 529 nm when excited to 495 nm). The assessments were done in cells and aqueous solutions. Under both circumstances, a much higher DCF fluorescence signal was found in Cu_2–*x*_S NIR-irradiated samples compared to the controls (neither NCs nor NIR), and only negligible DCF fluorescence was observed in Cu_2–*x*_S group (no NIR exposure). An increase in temperature was recorded not only as the NPs concentration increased but also as copper leakage took place. The detected absorbance is ascribed to ROS formation while the emission signals are caused by the cleavage of the acetate groups.

This leakage was proposed as the initiator behind ROS production. Under NIR light and tumor acidic environment, Cu(I) ions leaking from the NCs react with surrounding hydrogen peroxide to form Cu(II), hydroxide, and hydroxyl radicals similar to that produced in a Haber–Weiss reaction. This ROS production along with the increase in temperature leads to tumor cell destruction. The combination of both therapies enhanced the in vitro and in vivo therapy results. In the in vitro tests, B16 cells were treated with PEG-Cu_2*x*_S NPs, and exposed for 3 min to an 808 nm NIR laser with a power density of 2.3 W cm^−2^. All the experiments evidenced increasing cytotoxicity against B16 cells in a dose and time-dependent manner. After 48 h, the IC50 values (0.995 μg mL^−1^ Cu) for Cu_2–*x*_S -NIR were about 11 times lower compared to those obtained at 24 h for the exposed group (15.27 μg mL^−1^). Fluorescence microscopy revealed disruption of the cell cytoskeleton for the treated group, while the control group treatments did not cause any significant disruption. The therapeutic efficiency was further assessed in vivo in B16 subcutaneous tumor-bearing nude mice for the Cu_2–*x*_S-NIR treated group and an inhibition of 90% was observed compared to the control group. The therapeutic efficiency is ascribed to the combination of PTT and PDT, making Cu_2–*x*_S promising for cancer treatment. Wang’s findings have catalyzed the development of other dual PDT and PPT Cu_2–*x*_S agents and paved the way for research on the mechanism behind intracellular ROS production. In this spirit, Li et al*.* prepared 5-nm pegylated Cu_2–*x*_S NPs and studied their photothermal and photodynamic activities for the treatment of lung adenocarcinoma SPC-A-1 in vitro and in vivo. SPC-A-1 lung cancer cells incubated with PEG-Cu_2–*x*_S NPs were irradiated with an 808-nm laser (1 W cm^−2^ power density). The in vitro combined therapy caused approximately 70% reduction in cell viability, whereas in the control cells incubated with Cu_2–*x*_S NPs without laser irradiation, no decrease in cell viability was observed. In the in vivo combined therapy, the mice bearing SPC-A-1 tumors were injected with PEG-Cu_2–*x*_S NPs, followed by irradiation using an 808-nm laser, and the tumor volume was monitored for 2 weeks. Tumor suppression and delay in growth were observed for the mice group treated with NIR and PEG-Cu_2–*x*_S NPs under irradiation, which was ascribed to the synergistic effect of NPs. Tumor tissues were collected and histochemically stained with hematoxylin and eosin. For the treated group, nuclear pyknosis, cytoplasmic edema, and some leaking patches of eosinophils were observed in the tissues, which are indicative of tumor necrosis. A decrease in the expression of Ki-67, a nuclear non-histone protein frequently overexpressed in proliferating cells, further confirmed the effectiveness of the dual therapy. Through a fluorescent assay, ^1^O_2_ was ascertained as one of the ROSs produced for IR-activated Cu_2–*x*_S in tumor cells.

**Fig. 7:**
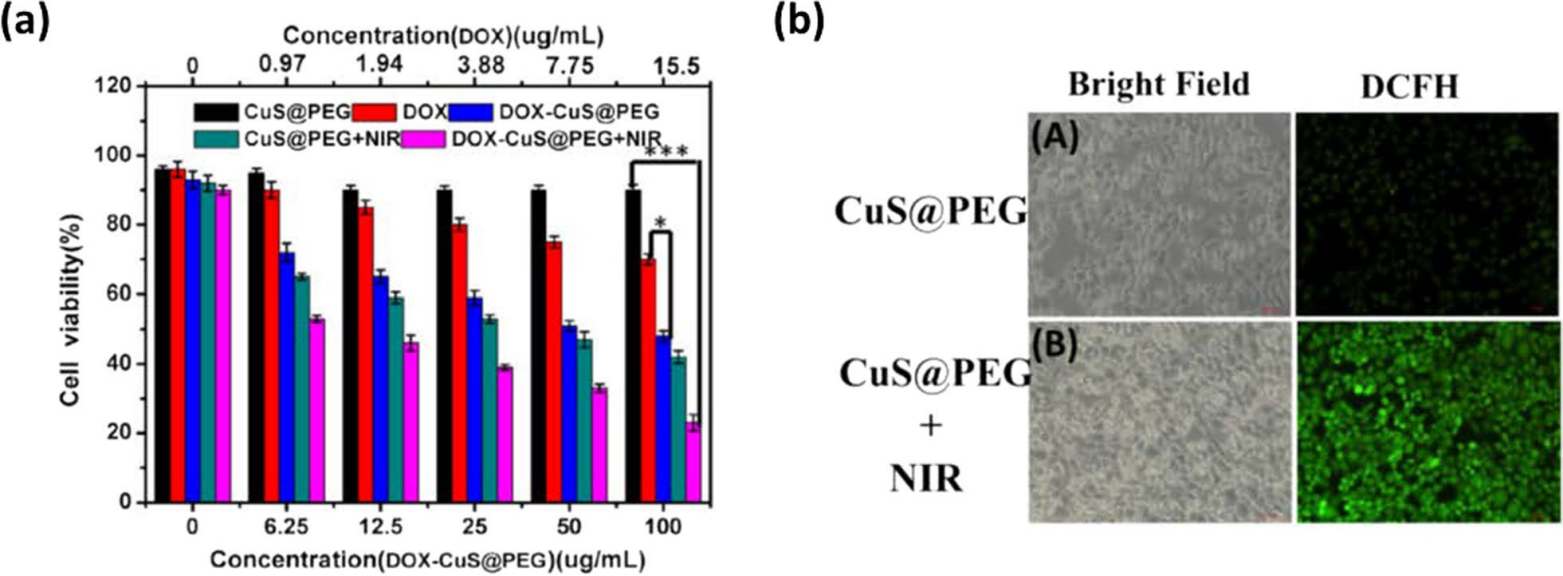
**a** Cell viability of HepG2 cells incubated with DOX-CuS@PEG nanocubes at different concentrations and subjected to NIR laser exposure (808 nm, 2 W cm^–2^) for 20 min. **b** Fluorescent images of DCFH stained HepG2 cells after curing with C_U_S@PEG with and without exposure of 808 nm light (2 W cm^–2^) for 10 min. Reproduced with permission from Ref. [75]. Copyright 2019, Elsevier, Amsterdam, Netherlands

To further improve the PTCE and ROS generation of Cu_2–*x*_S NPs, hollow-structured Cu_2–*x*_S nanocubes were developed following a Kirdenkall approach [75]. CuO nanocube and thioacetamide were used as precursor and sulfur source, respectively, under optimized reaction time and reagent concentrations. The particle size ranges from 250 to 300 nm. Given their hollow structure, Cu_2–*x*_S nanocubes (compared to spherical 100 nm Cu_2–*x*_S NPs) showed improved PTCE (30.3%) and good structural stability against laser exposure. This enhancement was related to the enhanced light reflex observed in hollow structures (mirror cavity effect). Moreover, given the porous cavity of the Cu_2–*x*_S hollow structure and the negative surface potential, doxorubicin, an anticancer drug, was loaded and showed a significant loading capacity of 15.49%. The in vitro synergistic effect of the chemotherapy associated to PTT and PDT displays enhanced cytotoxicity on HepG2 cancer cells, as shown in Fig. 7. ROS generation was assessed in vitro and in PBS via the DCFH-DA assay. Strong green fluorescence was observed for cells treated with NIR-Cu_2–*x*_S NPs. To detect the possible active ROSs, the author added two scavengers (p-benzoquinone and isopropanol) to the DCFH-DA PBS solutions. The addition of these scavengers induced a decrease in fluorescence signal, suggesting the formation of ·OH and ·O_2−_ radicals.

The authors also measured the fluorescence spectra of DCFH treated with Cu^+^, Cu^+^ (ethanediamine, a chelating agent of Cu ion), Cu^+^ (N_2_ bubble), and Cu^2+^ solution to further confirm the effect of Cu^+^ ions on ROS production. Only under the first condition, ROS generation was detected, which further confirmed the pivotal role of Cu^+^ in the photodynamic activity of Cu_2–*x*_S NPs. In addition, it was noted that an increase in temperature and immersion time considerably benefits the ROS production, which reinforces the PTCE relevance of these NPs. Based on these findings, the proposed mechanism for Cu-based PSs is as follows.

Firstly, Cu^+^ leaked from the NPs reacts with the surrounding O_2_ (oxidizes to Cu^2+^) through a single electron process

1 $${\text{O}}_{2} + {\text{Cu}}^{ + } \to {\text{Cu}}^{ + 2} + {\text{O}}_{2}^{ - } { }$$

Then, ·O_2_− can further react with the surrounding Cu^+^ to produce H_2_O_2_,

2 $${\text{O}}_{2}^{ - } { } + {\text{Cu}}^{ + } \to {\text{Cu}}^{ + 2} + {\text{H}}_{2} {\text{O}}_{2}^{ - }$$

This Cu^2+^ can react with H_2_O_2_ through the Haber–Weiss and Fenton reactions generating ·OH keeping the cycle between Cu^+^ and Cu^2+^ redox states,

3 $${\text{H}}_{2} {\text{O}}_{2} + {\text{Cu}}^{2 + } \to {\text{Cu}}^{ + } {\text{O}}_{2} {\text{H}} + {\text{H}}^{ + }$$

4 $${\text{H}}_{2} {\text{O}}_{2} + {\text{Cu}}^{ + } \cdot {\text{O}}_{2} {\text{H}} \to {\text{Cu}}^{ + } + {\text{O}}_{2} + {\text{OH}} + {\text{H}}_{2} {\text{O}}$$

Considering the abundant hole carriers in CuS nanomaterials, a second mechanism was proposed for ROS generation, which would be mediated through a reaction between holes and water molecules (photocatalysis). However, some questions remain open regarding the exact ROS production mechanism, which requires further study for the improvement of the therapy.

**Fig. 8:**
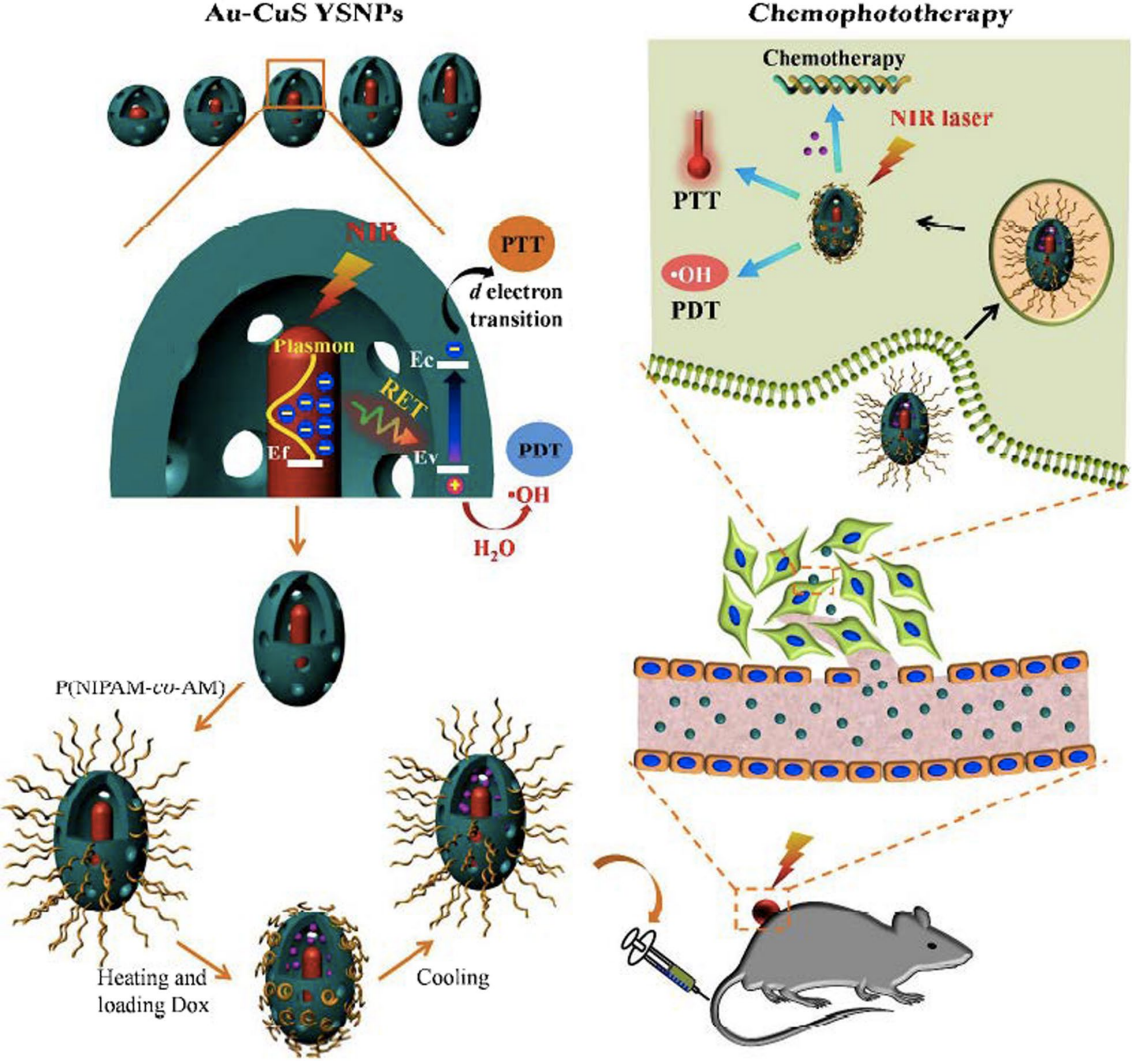
Graphical representation showing the improved therapeutic performance of Au-CuS YSNPs based on resonance energy transfer (RET) activation that can be used for chemo-, photothermal, and photodynamic combination therapy. Reproduced with permission from Ref. [77]. Copyright 2018, American Chemical Society, Washington, DC

In pursuit of developing CuS-based systems that exhibit similar synergistic photothermal and photodynamic effect on cancer cells, Huang et al. reported a yolk-shell structure of Cu_2−*x*_S and upconversion nanoparticles (UCNPs) [76]. The as-prepared UCNPs@CuS system showed higher generation of ROS species (such as hydroxyl radicals and ^1^O_2_) when compared to bare CuS counterparts. The PDT effect combined to an enhanced photothermal effect resulted in significant death of 4T1 murine mammary carcinoma cells under an irradiation of 808 nm. Likewise, Chang et al*.* developed a yolk-shell nanoparticles (YSNPs) of gold (Au) core@void@copper sulfide (CuS) shell (Au-CuS) for chemo-, photothermal, and photodynamic combination therapy of cancer [77]. The authors observed that this structure facilitates the incident light to be concentrated into nanoscale hotspots of plasmonic metal cores, when there is a match of the incident light wavelength with the LSPR absorption wavelength of the plasmonic metal core, which produces local electromagnetic field enhancement to induce a resonance energy transfer (RET) from the plasmonic metal to the semiconductor. The activation of RET process could be thereby used in plasmonic metal core@void@CuS shell YSNPs for improvement of both photothermal and photodynamic performance of CuS (see Fig. 8), as the authors indicated.

**Fig. 9:**
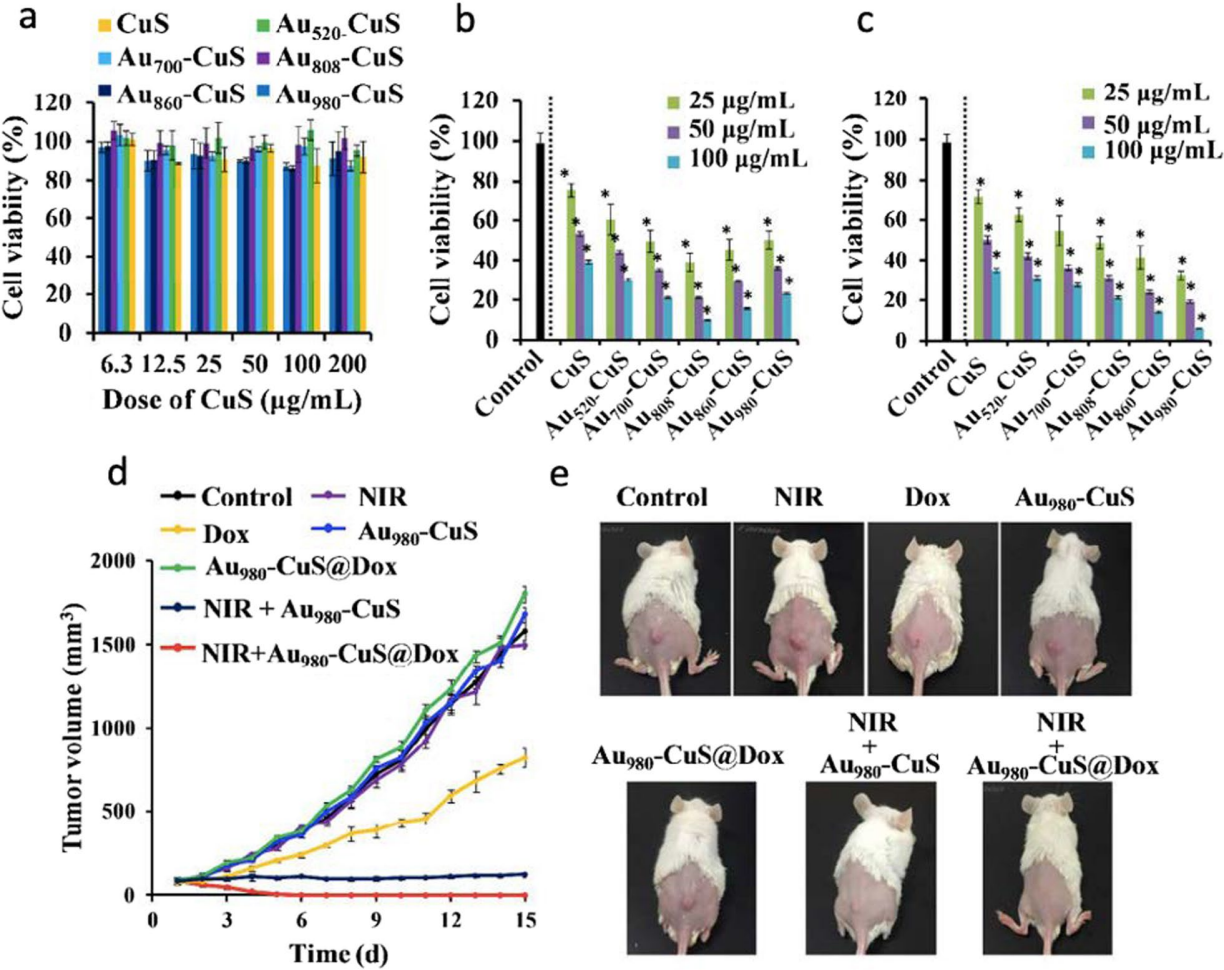
**a** Viability assessment of 4T1 cells exposed to different concentrations of NPs (CuS content) for 24 h. **b**, **c** Viability assessment of 4T1 cells treated with different concentrations of NPs for 6 h, followed by 5-min irradiation by an 808 nm (**b**) or 980 nm (**c**) laser at 0.75 W cm^−2^ and another 18 h of incubation. **d** Tumor growth curves of 4T1 tumor-bearing mice after IV injection with PBS, Dox, p-Au_980_-CuS, p-Au_980_-CuS@Dox in the presence (980 nm laser, 0.9 W cm^−2^, 5 min) and absence of irradiation. **e** Representative photos of tumor-bearing mice after treatment. Reproduced with permission from Ref. [77]. Copyright 2018, American Chemical Society, Washington, DC

RET activation was proved to significantly improve the photothermal performance to almost 50% compared to their bare counterparts (p-CuS HNPs and Au) and other physical mixtures. RET activation from Au core to CuS shell also enhanced the electron–hole pair in CuS shell, facilitating more radical formation thus resulting in superior photodynamic performance. Interestingly, no other ROS species such as ^1^O_2_ and ^**.**^O_2_^–^ were detected, which could be indicative of a type I PDT process that might be useful for the treatment of hypoxic tumors. To optimize and obtain the most effective RET process of Au-CuS YSNPs, the morphology of Au cores was varied from nanospheres to nanorods yielding different LSPR absorption peaks at 520, 700, 808, 860, and 980 nm. It was found that Au_808_ and Au_980_ induced the highest temperature elevation and ·OH production under 808 and 980 nm laser irradiation, respectively. Similarly, increasing of shell thickness reduced the RET efficiency. Through in vivo studies, the authors concluded claiming that p-Au-CuS YSNPs exhibit efficient tumor accumulation, effective tumor growth inhibition, excellent biocompatibility in 4T1 tumor-bearing mice, and superior photodynamic properties to treat cancer (see Fig. 9).

### Other Metal Sulfides

**Fig. 10:**
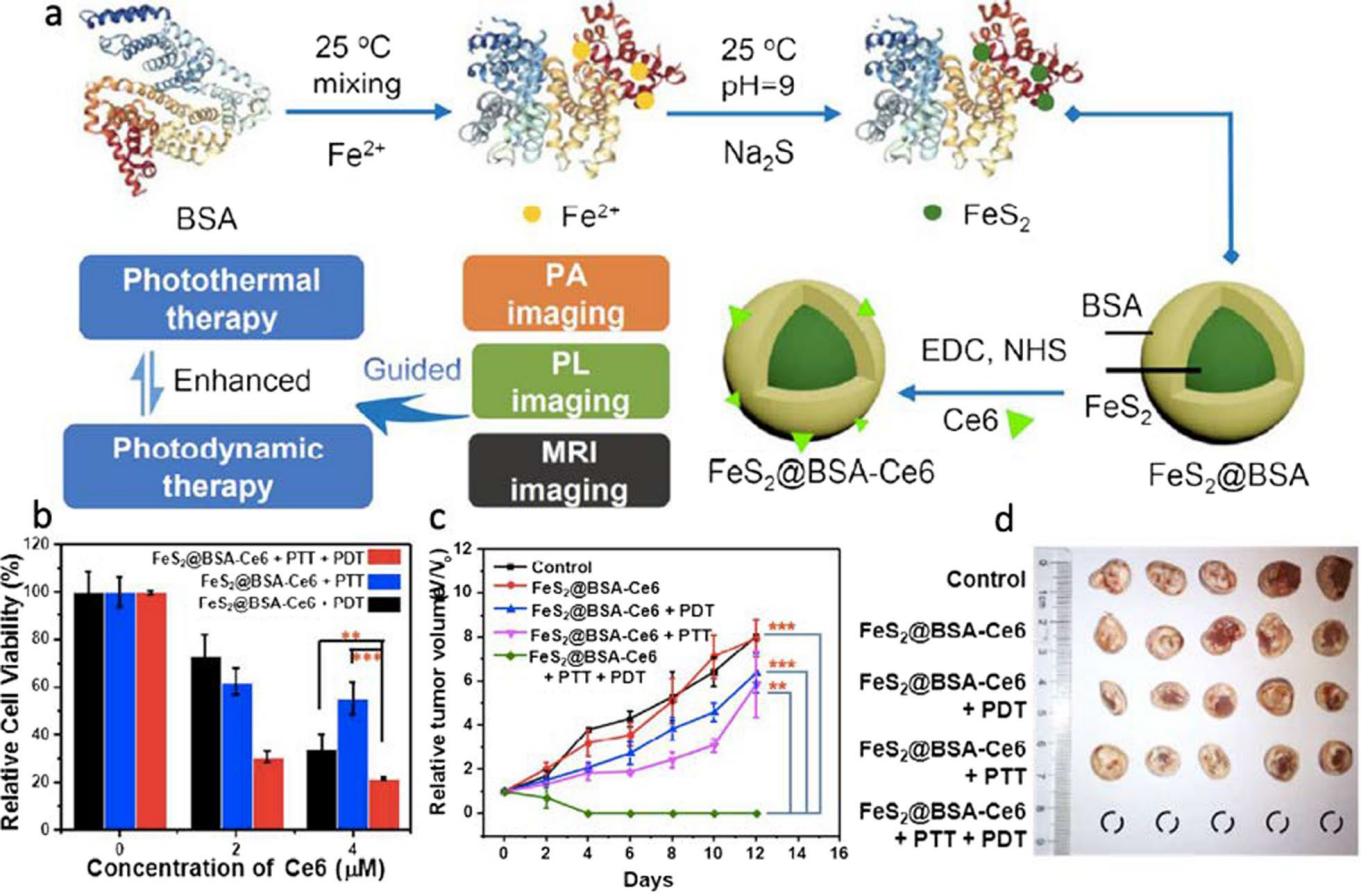
**a** Synthetic route and photodynamic effect of FeS_2_@BSA-Ce6 for PDT and PTT therapies. **b** Cells treated with FeS_2_@BSA-Ce6 and irradiated by a 660-nm lamp (5 mW cm^–2^, 30 min), 808-nm laser (0.8 W cm^–2^, 20 min) or both. **c** Relative tumor volume curves and corresponding **d** photograph of tumors collected from different groups (PBS-control, FeS_2_@BSA-Ce6, FeS_2_@BSA-Ce6 + PDT, FeS_2_@BSA-Ce6 + PTT, and FeS_2_@BSA-Ce6 + PTT + PDT). Reproduced with permission from Ref. [87]. Copyright 2018, American Chemical Society, Washington, DC

More recently, other nanostructured metal sulfides with unique physical and chemical properties and promising photodynamic response for cancer treatment have been reported. Among them, iron sulfide [78], silver sulfide [79, 80], bismuth sulfide [81, 82], and cobalt sulfide [83] stand out. Iron sulfide comprises iron and sulfur at different proportions and exists in different phases, such as Fe_1−*x*_S (pyrrhotite), FeS (mackinawite), FeS_2p_ (pyrite), Fe_3_S_4_ (greigite), Fe_9_S_11_ (smythite), and FeS_2m_ (marcasite) [78]. The phase, shape, and physicochemical properties of iron sulfide nanoparticles depend primarily on the iron content [78]. FeS_2_ is considered a non-toxic material and its production is cost-effective due to the high abundance of its elements [84]. Its unique magnetic properties, biocompatibility, biodegradability, and facile synthesis, make FeS_2_ attractive as a theranostic agent [85, 86]. Inspired by these excellent features, Jin et al*.* developed a nanodot system based on FeS_2_@BSA-Ce6 and evaluated its efficiency on murine breast cancer (4T1) cells (see Fig. 10) [87]. The authors found that FeS_2_@BSA-Ce6 nanodots induce significant cell death and slow tumor growth upon irradiation (660 nm xenon lamp at 5 mW cm^−2^ for 30 min), as shown in Fig. 10. Also, in efforts to merge PDT and PTT therapies using FeS_2_, Li et al*.* investigated the efficiency of FeS_2_@C-PEG yolk-shell nanostructures in both therapies [88]. They reported that both the particles excited by the light source and dissolved O_2_ played critical roles in producing ROS. The authors also conjugated the indocyanine green (ICG) photosensitizer to FeS_2_@C-PEG to enhance the PDT and PTT efficiency. It was observed that FeS_2_@C-PEG can oxidize water to form O_2_ under NIR exposure, which can in turn increase the therapy efficiency. Furthermore, the authors claimed that the Fenton reaction of Fe(II) makes FeS_2_ degrade intracellular H_2_O_2_ to produce more effectively ·OH and O_2_.

**Fig. 11:**
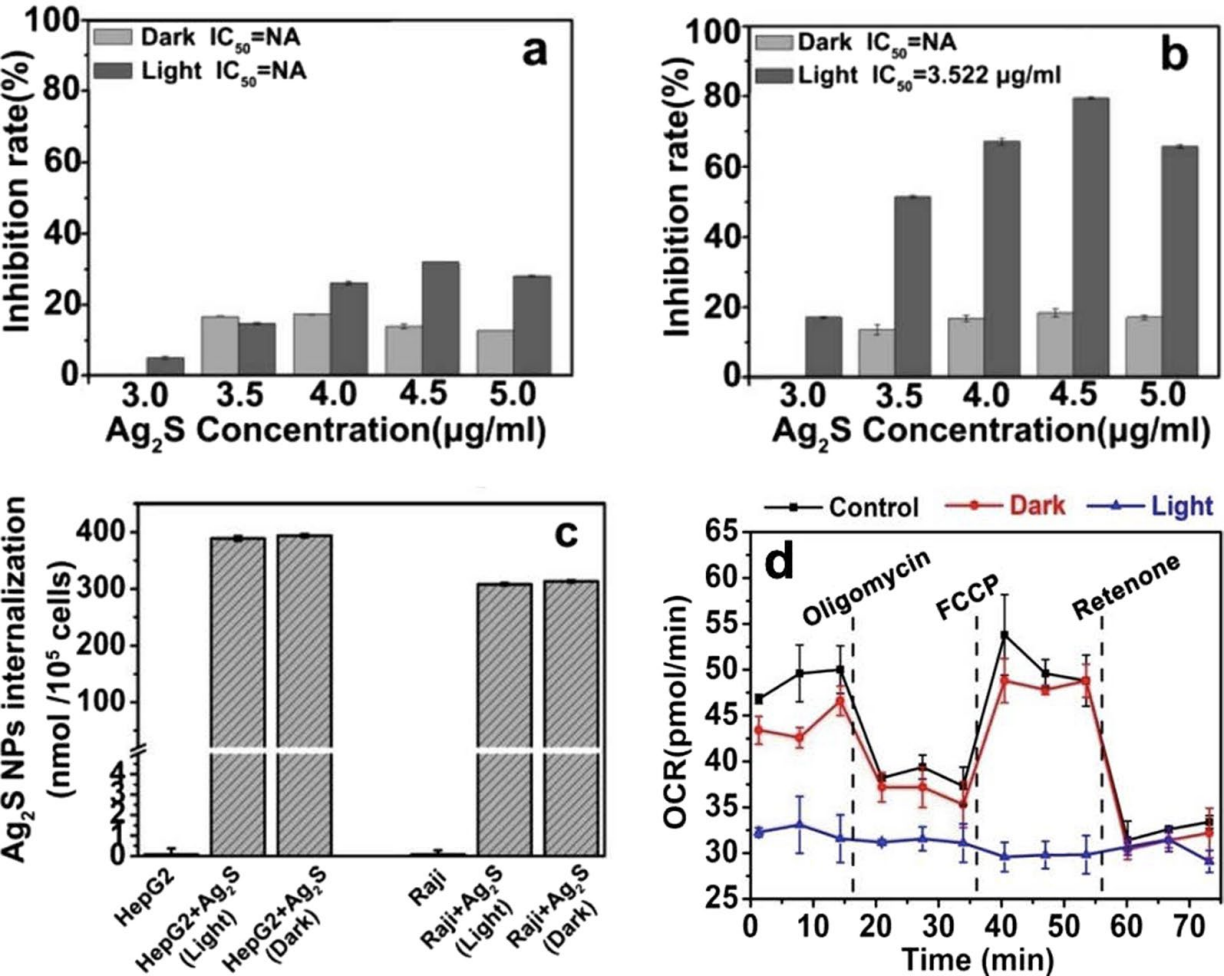
**a** Proliferative effects of **a** Hep G2 cells and **b** Raji cells treated with Ag_2_S NPs under dark and light irradiation for 72 h. **c** ICP-MS analysis showing Ag_2_S NP amounts internalized in Hep G2 and Raji cells under dark or light irradiation. **d** Cellular oxygen consumption rate (OCR) of Raji cells by treatment with Ag_2_S NPs under dark and light irradiation. Reproduced with permission from Ref. [89]. Copyright 2019, Royal Society of Chemistry, Washington, DC

**Fig. 12:**
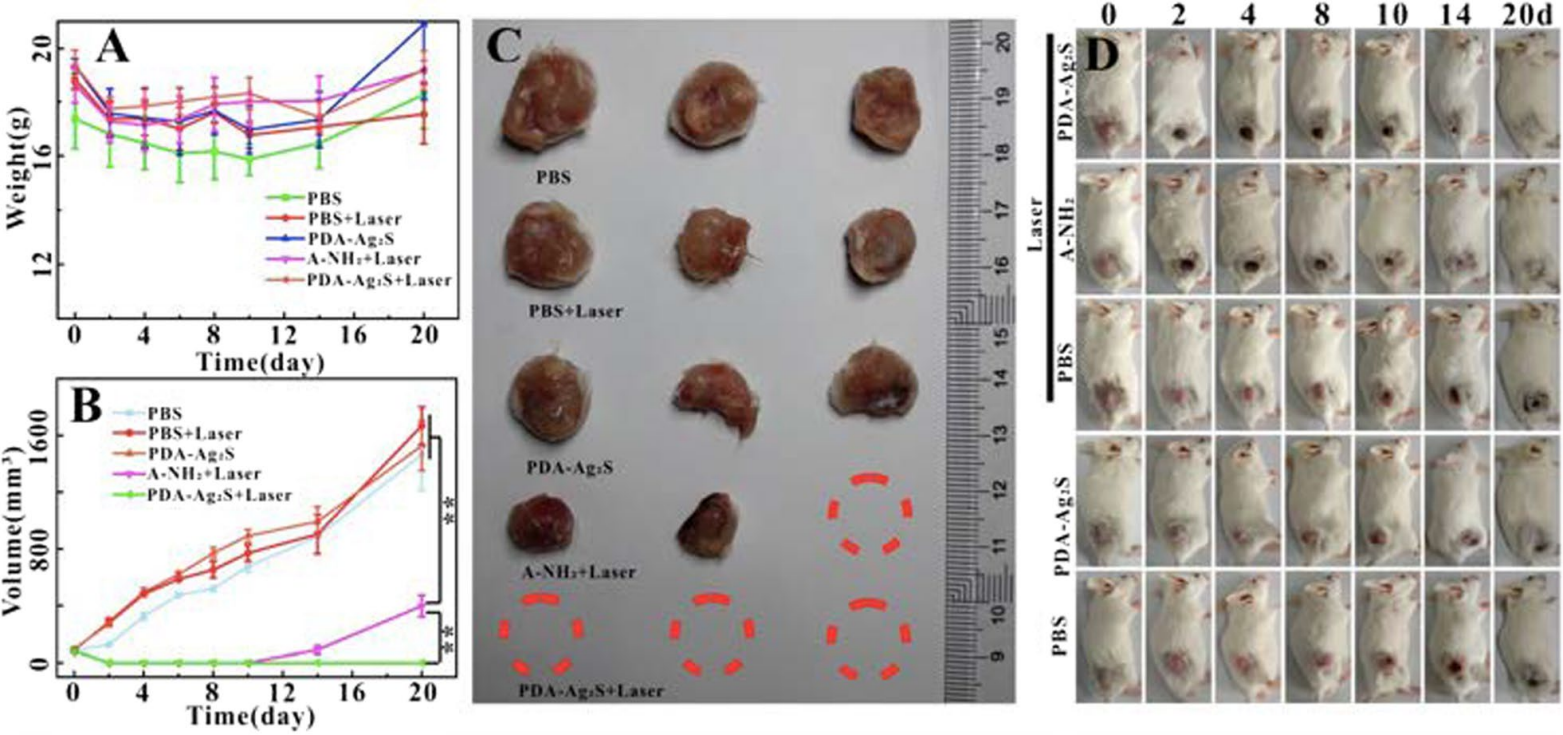
Tumor weight (**A**) and volume (**B**) change, tumor white-light (**C**), white light (**D**) after tumor irradiation (10 min, 808 nm, 1 W cm^−2^) in tumor bearing mice after injection of PBS, A-NH_2_ and PDA-Ag_2_S. Reproduced with permission from Ref. [90]. Copyright 2019, Chemistry Europe, European Chemical Societies Publishing

Another interesting nanostructured metal sulfide system is silver sulfide (Ag_2_S) that exhibits excellent biocompatibility, superior optical properties, and a wide range of applications, including bioimaging, fluorescent detection of molecules and metal ions, electronics, catalysis, and energy conversion. Recently, Ag_2_S NPs have been proposed to serve as PSs for PDT to treat more aggressive, chemoresistant and non-solid tumors. For instance, Wang et al*.* reported the synthesis of Ag_2_S NPs using bovine serum albumin (BSA) as a stabilizer to treat lymphoma [89]. In vitro studies confirmed that Ag_2_S NPs could significantly control the proliferation of human lymphoma cells compared to hepatoma carcinoma cells under light irradiation (see Fig. 11). The authors used a diode laser with a wavelength ranging from 400 to 600 nm (power of 0.4 W cm^−2^) to irradiate the samples for 10 h. They noticed that the strongest absorption peak occurred in the range of 200–600 nm indicative of the strong photoabsorption response of Ag_2_S NPs. Finally, Ag_2_S NPs can also induce both ROS accumulation in human lymphoma cells under light irradiation and significant disruption of energy metabolism (see Fig. 11). In a separate account, Cheng et al*.* reported the photodynamic therapy of Ag_2_S QDs and its enhanced regulation based on polydopamine (PDA) to treat mammary carcinoma (see Fig. 12) [90]. The authors modified the surface of Ag_2_S QDs with PEGylated phospholipids (DSPE-PEG_2000_-NH_2_) to generate ^1^O_2_ under irradiation of 808-nm NIR light. To further increase ROS production, Ag_2_S QDs were coupled with PDA (PDA-Ag_2_S) resulting in significant enhancement of PDT effect. In vitro studies showed identical PDT effects of Ag_2_S and PDA-Ag_2_S at longer wavelength under irradiation of 660 nm nm laser. Whereas in vivo therapeutic assays on 4T1 tumor bearing female mice revealed that PDA-Ag_2_S showed an improved PDT efficacy when compared to Ag_2_S (see Fig. 12). This new PS with longer absorption wavelength (deeper tissue penetration) and enhanced regulatory effect originated from PDA has enormous advantages to expand the application of PDT in tumor therapy.

**Fig. 13:**
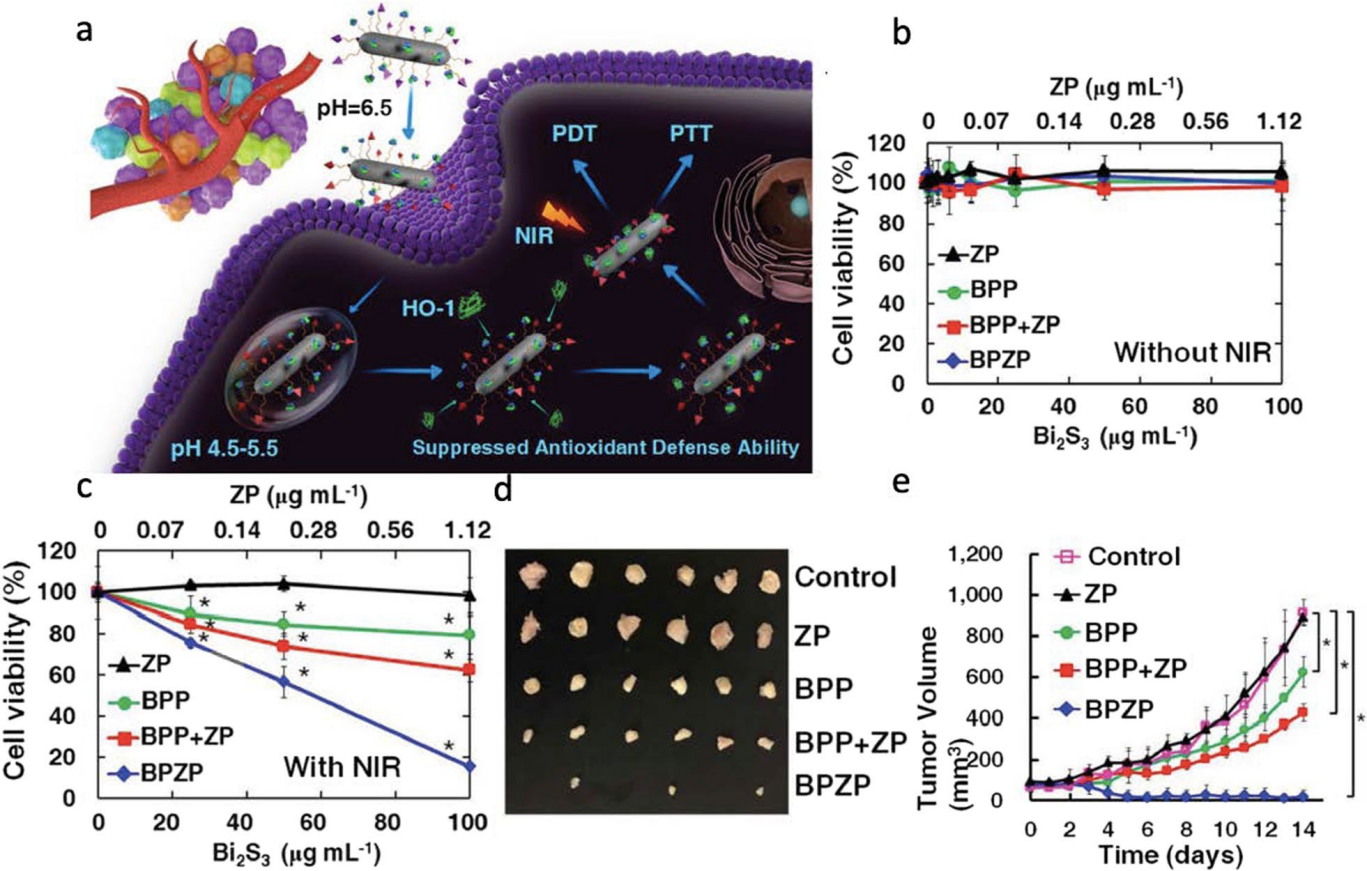
**a** Schematics illustrating the in vivo therapeutic process using a Bi_2_S_3_ nanorod-P(NIPAM-co-AM)-zinc protoporphyrin IX-Pep (BPZP) system through suppressing HO-1 activity and promoting NIR laser-irradiated ROS production after intravenous administration. **b**, **c** Viability of cells treated with various concentrations of ZP, BPP, BPP + ZP, and BPZP for 24 h with or without 808 nm laser irradiation (0.75 W cm^−2^, 10 min). **d** Representative photos of tumors dissected at 14 d post-treatment. **e** Tumor growth curves of mice IV injected with ZP, BPP, BPP + ZP, and BPZP (equivalent to 20 mg kg^−1^ Bi_2_S_3_ or 0.224 mg kg^−1^ ZP) with 808 nm laser irradiation (0.75 W cm^−2^, 5 min) for 14 d. Reproduced with permission from Ref. [92]. Copyright 2019, WILEY–VCH Verlag GmbH & Co. KGaA, Weinheim, Germany

In parallel, similar nanostructured metal sulfide systems have been reported to treat other types of cancer via PDT and PTT. In this regard, Faghfoori et al*.* developed a nanosystem based on bovine serum albumin-coated Bi_2_S_3_ (Bi_2_S_3_@BSA) NPs conjugated with methotrexate (MTX) to form Bi_2_S_3_@BSA-MTX NPs to study its anticancer effect on human colon adenocarcinoma [91]. *In vitro* chemo-radiation therapy findings revealed that the viability of treated cells with Bi_2_S_3_@BSA-MTX NPs is significantly lower than the cells treated with Bi_2_S_3_@BSA NPs. The apoptosis assay, without X-ray radiation, showed that Bi_2_S_3_@BSA-MTX NPs (at 300 μg mL^−1^) induced a significant percentage of cell death while Bi_2_S_3_@BSA-MTX NPs (at 100 μg mL^−1^), with X-ray irradiation, demonstrated a considerable rate of apoptosis, which confirmed the ability of the Bi_2_S_3_@BSA-MTX NPs as radio-sensitizer and their potential therapeutic efficacy in living organisms. To treat other types of cancer, Cheng et al. studied the computed tomography (CT) imaging-guided photodynamic and photothermal properties of bismuth sulfide (Bi_2_S_3_) nanorods (NRs) linked to zinc protoporphyrin IX (ZP) (BPZP) through a thermally retractable poly(N-isopropylacrylamide-co-acrylamide) (P(NIPAM-co-AM)) polymer, which were intravenously administrated to 4T1 tumor-bearing mice (see Fig. 13) [92]. The authors observed that the fast electron–hole recombination within low bandgap of Bi_2_S_3_ significantly precluded the photodynamic response. They also observed that the heat released from Bi_2_S_3_ NRs upon NIR laser irradiation could retract the polymer and drive ZP to the proximity of Bi_2_S_3_ NRs, which facilitates an efficient electron–hole separation in ZP and Bi_2_S_3_ NRs and leads to ROS generation. Mechanistically speaking, the ZP molecules play a crucial role in effectively binding to the active site of the HO-1 enzyme to suppress the cellular antioxidant defense ability, and in fostering the subsequent ROS injury in absence of IR irradiation. Also, such molecules can promote efficient electron–hole separation to enhance ROS generation by transferring NIR laser-induced holes from Bi_2_S_3_ to ZP. As concluding remarks, the authors stated that BPZP can accumulate into tumor, inhibit tumor HO-1 activity (see Fig. 13), and enhance NIR irradiated oxidative injury. BPZP can act as an effective and biocompatible antitumor nanosystem in PDT and PTT.

**Fig. 14:**
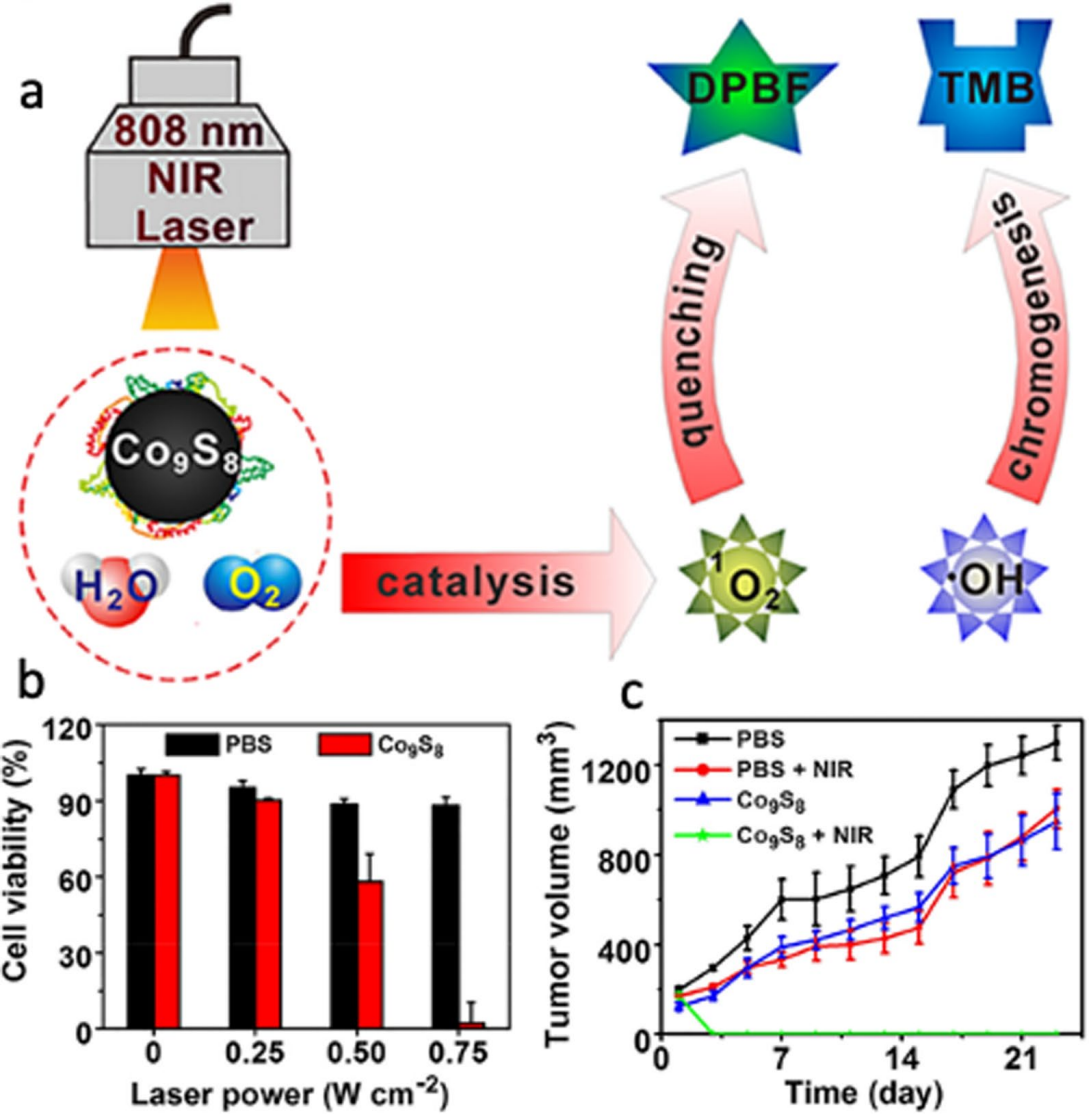
**a** Schematic illustration of the photocatalytic activity of Co_9_S_8_ nanodots upon NIR irradiation. **b**, **c** Cell killing and tumor inhibition capabilities of Co_9_S_8_ nanodots in PDT and PTT therapies. Reproduced with permission from Ref. [83]. Copyright 2018, American Chemical Society, Washington, DC

The last nanostructured metal sulfide system to mention is cobalt sulfide, which has intrinsic peroxidase-like activity useful in PDT to treat cancer. Cobalt chalcogenides are typically synthesized for NIR light activatable PTT due to their high photothermal conversion efficiency and broad optical absorption in the NIR region [93]. Cobalt sulfide ultrasmall NPs are also applicable for systemic circulation of theranostic agents and used as NIR responsive nanostructures for PTT/PDT therapies. There are however some challenges to synthesize biocompatible cobalt sulfide NPs, which demands to employ innovative surface modification strategies. To address these challenges, Lin et al*.* synthesized multifunctional cobalt sulfide nanodots (Co_9_S_8_ NDs) using an albumin-biomineralized approach for photocatalytic synergetic therapy with tumor multimodal imaging navigation (see Fig. 14) [83]. The synthesis was driven by breeding BSA with CoCl_2_ to form the Co^2+^-BSA complex in the presence of Na_2_S to trigger the nucleation of Co_9_S_8_ NDs. The authors used 1,3-diphenylisobenzofuran (DPBF) as a probe for ^1^O_2_, which can decrease the fluorescence intensity of DPBF due to oxidation reaction. They found that upon NIR irradiation (808 nm, 0.75 W cm^−2^) the NDs dispersed in aqueous solution at 100 μg mL^−1^ Co showed a marked time-dependent ^1^O_2_ production, and the DPBF fluorescence decreased ~ 40% in the presence of NDs. This suggests that the NDs can generate ^1^O_2_ when NIR irradiated. Also, NIR irradiation stimulated charge collection and separation at the surface thereby decreasing the recombination of photo-triggered charge carriers and accelerating the surface-dependent reactions, which can enhance the photosensitization of Co_9_S_8_ NDs. The results showed that Co_9_S_8_ NDs are photostable with photothermal conversion efficiency of 64%. Then the authors irradiated.

K7M2 cells (808 nm laser) at different power densities after incubation with the NDs (25 μg mL^−1^ Co) for 6 h. It was found that all NDs-treated cells were killed under NIR irradiation at 0.75 W cm^−2^ for 10 min (see Fig. 14). Based on these results, the authors investigated if this effective photocatalytic and photothermal effect can be translated into in vivo settings to treat tumors. To do this, the authors intravenously injected NDs into mice bearing K7M2 tumors, and then tumors were irradiated with 808 nm laser at 24 h post-injection. After 5 min of laser treatment, the surface temperature of tumors on NDs group reached about 60 °C, while less than 5 °C was observed for irradiated tumors on PBS group. After continuously monitoring for 21 days, the authors found that the tumor volume of the other groups showed a three–fivefold increase (compared to their original volume) and only NDs treated group exhibited tumor growth inhibition (see Fig. 14). Co_9_S_8_ NDs can under NIR irradiation generate ROS to inducing cancer cell death, completely suppressed the tumor growth and even eradicated the tumor. The authors concluded stating that this biomimetic strategy could represent a significant progress for enhanced PTT/PDT synergistic therapy with single NPs under NIR irradiation.

Even though PDT shows certain limitations associated to limited light-penetration depth (for visible light source < 1 mm), MTSs acting as next-generation PSs and phototherapy agents have the potential to address these issues. They possess strong absorption in the NIR region (around 700–1100 nm), high extinction coefficients, and versatile surface chemistry. Biocompatible MTS PSs can also serve as carrier nanoplatforms to address issues related to low solubility, poor tumor selectivity and undesirable pharmacokinetics of traditional PSs. MTS PSs also exhibit promising physicochemical properties, including Fenton catalysis, light conversion, radiation enhancement, and immune activation, and synergistic antitumor properties, which are used in cancer therapy.

## Conclusions and Outlook

Over the past decades, PDT has emerged as a promising technique for cancer therapy. Despite its wide application, PDT poses several challenges related to low tissue penetration of excitation light, instrumentation, tissue oxygenation, and inherent photo-biochemical properties of PSs that limit its clinical efficacy. As a result, it is mainly employed for the treatment of superficial tumor or lesion in recent clinical settings. While some studies in PDT attempted to utilize diode laser light and design NIR light-triggered PSs to overcome tissue penetration-based concerns, issues of innate toxicity and hydrophobicity of PSs remain profoundly challenging [94–96].

These challenges are topics of active research, and the advances in nanomedicine have led to brisk progress in the development of sophisticated PSs that promise to overcome such innate biochemical properties to some extent. Engineered nanomaterials offer unparalleled advantages, endowing enhanced chemical activity and stimulating photo-stimulated reaction at low power irradiation. Nanostructures are adsorbed by cytomembranes and lead to cell oxidation by protein denaturation, DNA damage, and ROS generation. Moreover, the size, morphology, and surface of nanostructures should be optimum to modify their ROS generation efficiency. In pursuit of high-performance nanostructured PSs, metal-based nanoparticles (e.g., gold nanostar and silver nanoparticles), metal–organic hybrid materials (e.g., TiO_2_ and ZnO nanoparticles), hollow mesoporous silica nanoparticles, and MTSs, were reported [97–101].

Nanostructured MTSs can be used as phototherapy agents due primarily to their strong absorption in the NIR region, high extinction coefficients, versatile surface chemistry, high fluorescence, magnetism, structural, and thermal stability. MTSs are easily obtained through low-cost synthetic methods (compared to other NIR absorbing metal NPs), which can be scaled up to advance the reproducibility thereof. Investigations also show that MTSs can be utilized as PSs for PDT and nanocarriers for drug delivery due to their loading capacity, low degradation, long-time period, smart targeting, and formulated release. Besides their NIR absorbing capability due to metal-like plasma oscillation near NIR region, MTSs exhibit excellent photothermal conversion efficiency under NIR irradiation, and currently are under extensive research. In the present review, different kinds of non-toxic nanostructured MTS PSs, including molybdenum disulfide, zinc sulfide, copper sulfide, iron sulfide, silver sulfide, bismuth sulfide, and cobalt sulfide, were comprehensively introduced and investigated for PDT, PTT, and chemotherapy applications. In these investigations, MTSs were typically coated with biocompatible polymers to mitigate the dark toxicity and to facilitate the interpenetration of cells and increased cell uptake under NIR irradiation. Among all their excellent properties, nanostructured MTSs were found to exhibit high absorption, Fenton reaction catalysis, innate photothermal and photodynamic response, controlled ROS production upon NIR irradiation, light source power dependent heat generation, and desired PDT efficiency. In most of these systems, photothermally enhanced PDT synergistic effect to treat solid tumors, induced photoablation of tumor cells and tissues, antitumor immune response, and great cell killing, and tumor inhibition capabilities were reported.

Despite all this progress, several persuading results and plenty of challenges (e.g., insignificant water solubility, photostability, and extended maintenance in tissues) limit the photodynamic clinical applications of MTSs. Biocompatibility, formulation, synthesis methods, and reliability of nanostructured MTSs must be carefully considered to further reduce the dark toxicity; improve the biodistribution, pharmacokinetics, clearance (after their therapeutic action), and discrimination between tumor and healthy tissues; and increase the upconversion efficiency. Functionalization of MTSs with specific target molecules to favor tumor accumulation should be also studied along with their degradation and metabolism. In this review, one of the main common limitations observed was the high laser power intensity, which in most cases was significantly higher than the clinically approved levels further restraining clinical translation. As for dose-limiting systematic toxicity of MTSs, no clear consensus has settled at least from the in vitro (mostly) and in vivo data covered in this review. In general, extensive research is continuously required to increase the PDT effectiveness and address these challenges. Taken altogether, to make PDT an interventional treatment and frontier method to fight against cancer, a critical and honest knowledge transfer from the different PDT-involved fields is desperately needed, with experts on this multidisciplinary topic effectively discussing the main hurdles and realistic opportunities.

## Data Availability

Not applicable.

## Abbreviations

PDT: Photodynamic therapy
PS: Photosensitizer
ROS: Reactive oxygen species
PEG: Poly(ethylene glycol)
^1^O_2_: Singlet oxygen
^3^O_2_: Molecular oxygen
PTT: Photothermal therapy
MTS: Metal transition sulfide
NMC: Nanostructured metal chalcogenide
S_1_: Excited singlet state
T_1_: Triplet excited state
NIR: Near infrared
LSPR: Localized surface plasmon resonance
PTCE: Photothermal conversion efficiency
RET: Resonance energy transfer
PDA: Polydopamine
BSA: Bovine serum albumin
MTX: Methotrexate
DPBF: 1,3-Diphenylisobenzofuran
EPR: Enhanced permeability and retention
CT: Computed tomography
QD: Quantum dot
ND: Nanodot
NP: Nanoparticle
NR: Nanorod
UCNP: Upconversion nanoparticle
FA: Folic acid
TEM: Transmission electron microscopy
